# Generation of CCR4/CD7 Bispecific CAR‐T Cells Resistant to Fratricide and Exhaustion

**DOI:** 10.1002/advs.202521443

**Published:** 2026-02-26

**Authors:** Sile Li, Yuanxin Li, Asif Rashid, Hong Kee Tan, Shing Chan, Man Yan Hui, Wilson Yau Ki Chan, Kee See Lam, Yinping Liu, Wenwei Tu, Wing Leung

**Affiliations:** ^1^ Department of Paediatrics and Adolescent Medicine Li Ka Shing Faculty of Medicine The University of Hong Kong Hong Kong Hong Kong SAR P. R. China; ^2^ Department of Paediatrics and Adolescent Medicine Hong Kong Children's Hospital Kowloon Hong Kong SAR P. R. China; ^3^ Children's Blood and Cancer Centre KK Women's and Children's Hospital SingHealth Duke‐NUS Singapore Singapore

**Keywords:** cancer immunotherapy, CAR T‐cell therapy, dual‐targeted therapy, hematological disease, t‐cell malignancies

## Abstract

The use of chimeric antigen receptor (CAR) T‐cell therapy for T‐cell malignancies is limited by fratricide, antigen‐escape, lack of functional endurance and adverse events such as multilineage cytopenia. To address these limitations, we developed a simple single‐step CD7‐depletion process followed by transduction with a lentiviral vector encoding a CCR4/CD7 bispecific CAR. CD7‐negative (CD7N) CCR4/CD7 CAR‐T cells could expand without experiencing fratricide, unlike the bulk CCR4/CD7 CAR‐T cells. The CD7N CAR‐T exhibited robust cytotoxicity against malignant T‐cell lines with heterogeneous CD7 and CCR4 expression in vitro and in vivo. Incorporation of EGFRt in the CAR construct allowed elimination by cetuximab in case of adverse events, whereas inclusion of c‐Jun in the construct reduced functional exhaustion after repeated tumor challenges in vitro. In comparison to non‐transduced CD7N cells and Bulk CAR‐T cells, scRNA‐seq analysis of CD7N CAR‐T cells revealed a unique AQP3+ CD4+ T‐cell subset following exposure to tumor cells. This cell subset exhibited broad activation of the Src/Ras/ERK and Bcl‐2 pathways, high levels of *SOS1* and *KLF2* expression, and specific ligand‐receptor interactions within the tumor necrosis factor superfamily. Collectively, these results suggest that further clinical development of CCR4/CD7 bispecific CD7N CAR‐T cells is warranted, including the AQP3+ subset with *SOS1*/*KLF2* modulation.

## Graphical abstract

The applications of CAR T‐cell therapy in T‐cell malignancies face limitations such as fratricide, effector‐cell exhaustion, and antigen‐escape. Herein, we developed fratricide‐ and exhaustion‐resistant CAR‐T cells that targeted CCR4 and CD7 simultaneously, with optional EGFRt safety switch. Additionally, scRNA‐seq unveiled new molecular targets, which might further enhance CCR4/CD7 CAR‐T therapy for T‐cell neoplasms.

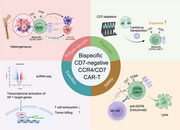

## 1 Introduction

Lymphoid neoplasms of B‐ or T‐cell origin are the fourth most common malignancy and the sixth leading cause of cancer death in the United States [1], as resistance to chemotherapy is common [2, 3]. Recently, chimeric antigen receptor (CAR)‐T cell therapies have demonstrated remarkable efficacy for the treatment of chemoresistant B‐cell malignancies, such as those targeting CD19 or BCMA [4]. CAR molecules guide engineered T lymphocytes to target antigens expressed by tumor cells by binding to its single‐chain variable fragment (scFv). Unlike traditional TCR‐mediated recognition, CAR‐T cell‐mediated signaling is not restricted by major histocompatibility complex (MHC) presentation. This allows CAR‐T cells to directly recognize, kill, and proliferate in response to tumor cells [4]. CAR‐T cell therapy has also achieved encouraging results in early‐phase studies of T‐cell malignancies [5, 6, 7, 8], with complete remission rates >90% for autologous CD7 CAR‐T [9]. CD7 has emerged as a promising therapeutic target for T‐cell malignancies because of its distinctive expression pattern on malignant leukemic cells. It is frequently detected in immature T‐cell malignancies, such as ETP‐ALL, which typically lack CD5 and CD1a expression [10, 11]. In addition, CD7 can still be identified during the minimal residual disease stage in T‐ALL patients following treatment [12, 13]. The effectiveness of CD7 CAR‐T cell therapy has been validated in multiple clinical trials [6, 7, 8, 14].

However, as CAR‐T applications expand from B‐cell tumors to T‐cell tumors, significant barriers remain; some barriers are specific to T‐cell targets, while other challenges pertain to all B‐cell and T‐cell targets. One specific barrier for T‐cell targeting is that the antigens (e.g., CD7) are often shared between normal T cells and cancer cells, resulting in CAR‐dependent self‐cytotoxicity (fratricide) [15]. Challenges that are shared among B‐ and T‐cell targeting include adverse events such as cytokine release syndrome (CRS) or second malignancies, and cancer recurrence due to antigen‐escape or CAR‐T exhaustion [4]. Among the 200 recipients of CD7 CAR‐T therapies in a systematic review, the incidence of CRS was high at 94% [16]. Rates of antigen‐negative relapses also appeared to be high. In a phase 2 study of CD7 CAR‐T, 13 of the 60 patients relapsed, with 5 of the 13 (38.5%) lost CD7 expression [14]. In a smaller study of 20 patients with long‐term follow‐up, 4 of the 6 relapses (67%) had documented loss of CD7, either because of frameshift or missense mutations in the *CD7* gene [17]. In another study, disease progression has also been observed due to minor populations of CD7‐negative blasts pre‐existed before CAR‐T therapy [8]. While CD7 is frequently overexpressed in immature T‐lymphoblastic leukemia/lymphoma, it is typically absent in mature T‐cell malignancies, such as adult T‐cell leukemia/lymphoma (ATLL), cutaneous T‐cell lymphoma (CTCL), and peripheral T‐cell lymphoma (PTCL), which are generally incurable [18]. CCR4 is a G protein‐coupled chemokine receptor characterized by seven transmembrane domains, which specifically recognizes the chemokines CCL17 (also known as TRAC) and CCL22 (also known as MDC) [19]. In contrast to CD7, CCR4 is highly expressed across various mature T‐cell neoplasms, including ATLL (88.3%), CTCL (31.5% to 91.6%, averaging 61.9%) [20, 21], and PTCL (31.3%) [22]. As CTCL progressed in longitudinal studies, a loss of CD7 expression [23], and an increase in CCR4 expression [24], were frequently observed. Accordingly, dual targeting of CD7 and CCR4 may mitigate antigen‐escape during CD7‐directed CAR‐T therapy and extend the therapeutic applicability to both immature and mature T‐cell neoplasms.

To address the major challenges of CAR‐T therapy for T‐cell malignancies, we first generated a novel tandem CAR that targeted both CD7 and CCR4 [8, 25, 26, 27]. Second, a simple CD7 cell‐depletion step was introduced to limit fratricide. Third, the CAR‐T functional endurance was improved by overexpression of c‐Jun. Fourth, CAR‐T elimination was made possible in case of adverse event by the inclusion of a truncated epidermal growth factor receptor (EGFRt) suicide domain. Finally, scRNA analyses were performed to further elucidate the CAR‐T biologic properties to identify potential pathways for therapeutic augmentation.

## 2 Results

### 2.1 Preparation of Fratricide‐resistant Immune Effector Cells

**FIGURE 1:**
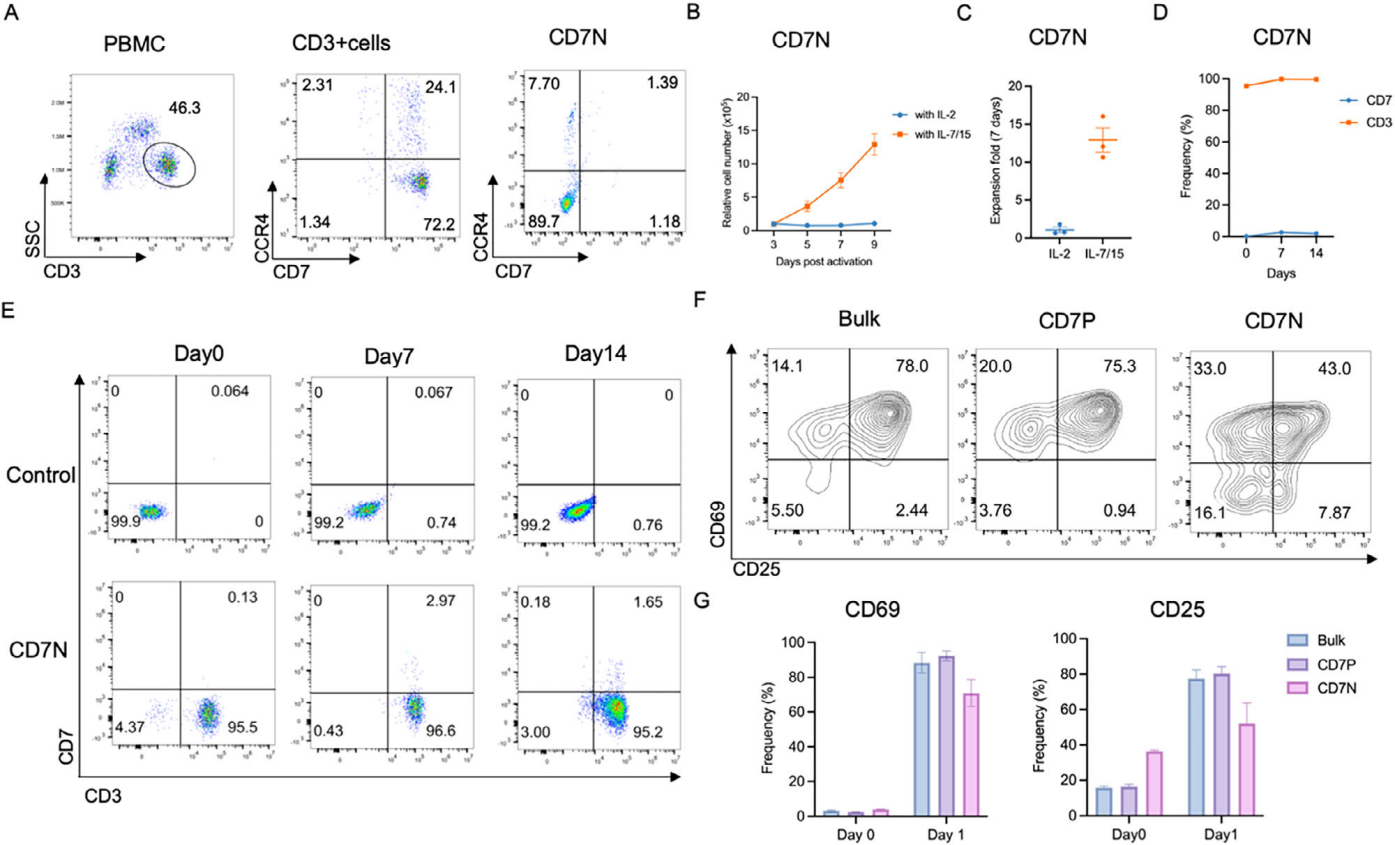
Phenotype and proliferation of CD7‐depleted cells (A) Representative dot plots showing CCR4 and CD7 expression on CD3+ T cells of whole blood from healthy donors and expression of CD7 and CCR4 on cells in the CD7‐negative fraction (CD7N) after immunomagnetic CD7‐depletion assessed using flow cytometry. (B,C) Proliferation and fold expansion of CD7N T cells cultured with IL‐2 or IL‐7/IL‐15. Proliferation data are presented as mean ± SEM. (D,E) Representative dot plots illustrating CD3 and CD7 expression on CD7N T cells during the culture process. Isotype controls were used for staining control CD7N T cells. (F,G) Expression of activation markers, CD69 and CD25, on T cells from the CD7N fraction or CD7‐positive (CD7P) fraction after CD7‐depletion before and after 24 h of activation. Expression on Bulk T cells is also shown. These markers were measured by flow cytometry. Results are presented as mean ± SD from three independent donors.

To develop CCR4/CD7 bivalent CAR‐T, it is not known whether a single‐step CD7‐depletion of peripheral blood mononuclear cells (PBMCs) will be sufficient to avoid fratricide or co‐depletion of CCR4/CD7 is necessary. Among PBMCs, the majority of CD3‐positive cells were CD7‐positive (94.6% ± 0.68%), while 19.96% ± 3.73% of CD3‐positive cells expressed CCR4 (Figure 1A; Figure S1A). After single‐step immunomagnetic CD7‐depletion, we found that large population (median 85%) of the cells in the CD7‐negative fraction (hereafter called CD7N) were CCR4‐negative (Figure 1A), suggesting that further depletion of CCR4 might not be necessary for subsequent clinical development to avoid cell loss and additional cost. These CD7N cells exhibited robust expansion when cultured with interleukin‐7 (IL‐7) and interleukin‐15 (IL‐15), in contrast to culture with interleukin‐2 (IL‐2) (Figure 1B,C). The CD7N cells maintained high levels of expression of CD3 but not CD7 throughout the 14‐day culture period (Figure 1D,E). After 24‐h of activation by the CD3 and CD28 agonists TransAct, CD69 and CD25 were upregulated from an average of 4.1% (± 0.1%) and 36.4% (± 0.6%) to 70.92% (± 7.8%) and 52.2% (± 11.6%) of the CD7N cells, respectively. Although these levels of upregulation are less than those in the CD7‐positive (CD7P) fraction after CD7‐depletion and in the unselected (bulk) T cells (Figure 1F,G), these observations suggested that the majority of CD7N cells could be activated by CD3/CD28 agonists for subsequent CAR vector transduction [28].

### 2.2 Individual CAR Activity and Safety of CCR4 Targeting

**FIGURE 2:**
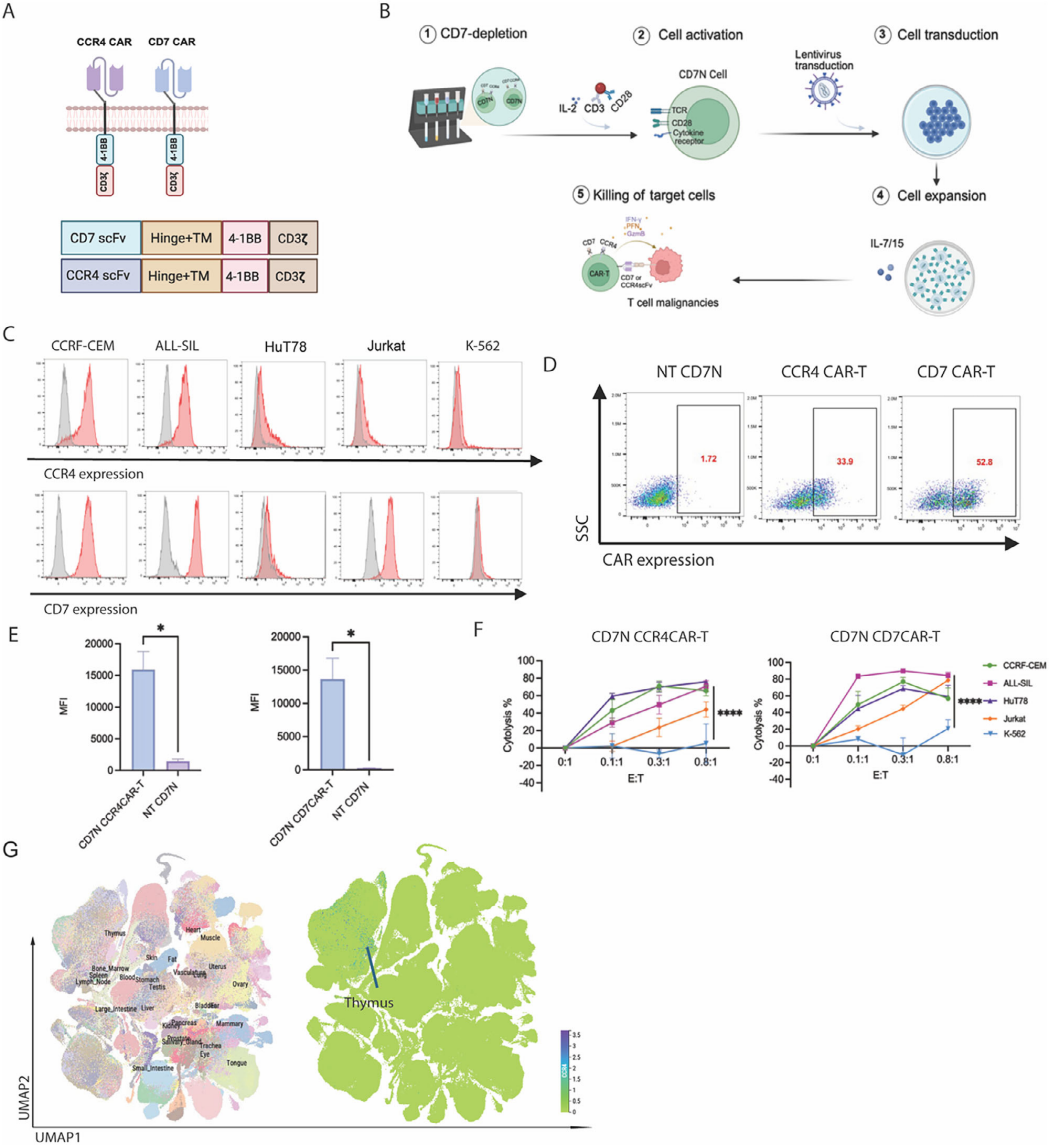
Activity of single‐targeting CD7N CAR‐T cells (A) Schematic of CCR4 CAR and CD7 CAR constructs. (B) A diagram summarizing the manufacturing process of monospecific CAR‐T. (C) Flow cytometry analysis of CCR4 and CD7 expression was performed in T‐lineage cell lines CCRF‐CEM, ALL‐SIL, HuT78, and Jurkat, using the myeloid cell line K‐562 as a negative control, and results were compared with isotype controls. (D,E) Representative dot plots and mean fluorescence intensity (MFI) showing CAR expression on non‐transduced (NT) CD7N cells or on CD7N cells expressing either CCR4 or CD7 CAR. Results are presented as mean ± SEM. ^*^ *p* < 0.05, unpaired 2‐ tailed *t*‐test. (F) Cytolytic activity of CD7N cells expressing either CCR4 or CD7 CAR against CCRF‐CEM, ALL‐SIL, HuT78, Jurkat, and control K‐562 after 24 h of co‐culture (*n* = 3). Data are presented as mean ± SEM. ^****^ *p* < 0.0001, two‐way ANOVA. (G) The UMAP visualization displays organ and tissue annotations along with CCR4 expression patterns across 1135218 cells derived from healthy human samples in the Tabula Sapiens scRNA‐seq dataset.

To validate the specificity and activity of each individual scFv and CAR (Figure 2A), we generated single‐targeting CAR‐T cells (Figure 2B) and tested their activity against cancer cell lines exhibiting a spectrum of CD7 and CCR4 expression (Figure 2C). CAR expression was determined by recombinant human CD7 protein and protein L binding (Figure 2D,E). Both types of single‐targeting CD7N CAR‐T cells demonstrated considerable cytotoxicity against T‐cell lines CCRF‐CEM, ALL‐SIL and HuT78, despite low effector‐to‐target (E:T) ratio of <1:1, when compared with negative control K‐562 cells (Figure 2F). Jurkat cells, which were CD7 bright but CCR4 dim, were susceptible to cytotoxicity from T cells with anti‐CD7 CAR but much less with anti‐CCR4 CAR. Collectively, these findings confirmed the general activity and specificity of each of the single‐targeting CAR.

CD7 has shown an excellent safety profile in multiple clinical studies. Prior to constructing the bispecific CAR, we evaluated the safety characteristics of CCR4 using the Tabula Sapiens single‐cell RNA sequencing dataset, a comprehensive human reference atlas covering 24 tissues and organs from healthy individuals [29]. The results showed that CCR4 expression was primarily restricted to the thymus, but expression levels remained low (Figure 2G).

### 2.3 Generation and Characterization of CCR4/CD7 Bispecific CAR‐T Cells

**FIGURE 3:**
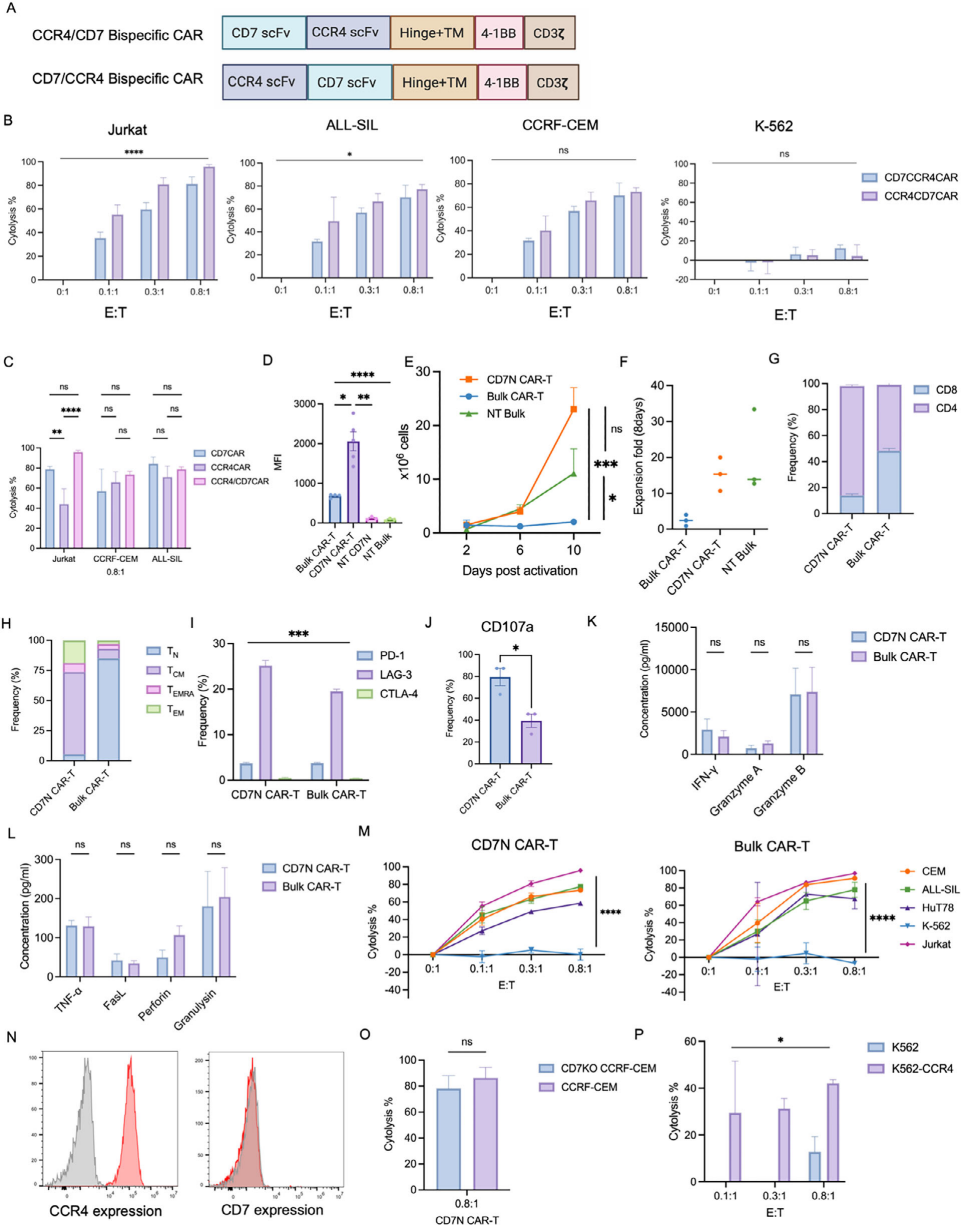
Generation and characterization of CCR4/CD7 bispecific CAR‐T cells. (A) Schematic of the CCR4/CD7 bispecific CAR and CD7/CCR4 bispecific CAR construct. (B) Cytotoxic effects of CD7‐negative CCR4/CD7 bispecific CAR‐T and CD7/CCR4 bispecific CAR‐T cells were assessed against Jurkat, ALL‐SIL, CCRF‐CEM, and control K‐562 cells following 24 h of co‐culture (*n*  =  3). Results are expressed as mean ± SD. ns denotes no significance, ^*^ *p* < 0.05, ^****^ *p* < 0.0001, based on two‐way ANOVA. (C) Cytotoxic activity of CD7N cells expressing CCR4 CAR, CD7 CAR, or the CCR4/CD7 bispecific CAR was evaluated against CCRF‐CEM and ALL‐SIL cells after 24 h of co‐culture (*n*  =  3). Data are shown as mean ± SD. ns indicates no significance, ^**^ *p* < 0.01, ^****^ *p* < 0.0001, determined by two‐way ANOVA. (D) MFI of CAR expression on CD7N CAR‐T cells and Bulk CAR‐T cells, compared to non‐transduced (NT) CD7N T cells and Bulk T cells. Results are presented as mean ± SEM. ^*^ *p* < 0.05, ^**^ *p* < 0.01, ^****^ *p* < 0.0001 using one‐way ANOVA. (E,F) Proliferation kinetics and fold expansion of CD7N CAR‐T, Bulk CAR‐T, and NT Bulk T cells over time. ns no significance, ^*^ *p* < 0.05, ^***^ *p* < 0.001, two‐way ANOVA. (G) CD4/CD8 ratio in CD7N CAR‐T and Bulk CAR‐T cell populations on day 10. Data are presented as mean ± SEM from three donors. (H) The frequency of naïve (T_N_), central memory (T_CM_), effector memory (T_EM_), and TEMRA(T_EMRA_) subsets in CD7N CAR‐T and Bulk CAR‐T cells measured by flow cytometry on day 10. Data are presented as average from three donors. (I) Expression of immune checkpoint receptors on CD7N CAR‐T and Bulk CAR‐T cells, assessed by flow cytometry on day 10. The results are shown as mean ± SEM from three donors (*n* = 3). ^***^ *p* < 0.001, two‐way ANOVA. (J) Degranulation of CD7N CAR‐T and Bulk CAR‐T cells after 4 h of co‐culture with Jurkat cells (*n* = 4). Data are presented as mean ± SEM for three donors. ^*^ *p* < 0.05, unpaired 2‐ tailed t‐test. (K,L) Cytokine release by CD7N CAR‐T and Bulk CAR‐T cells after 24 h of co‐culture with Jurkat cells, as measured by LegendPlex assay (*n* = 3). Data are presented as mean ± SEM, ns no significance, two‐way ANOVA. (M) Cytolytic activity of CD7N CAR‐T and Bulk CAR‐T cells against CCRF‐CEM (CEM), ALL‐SIL, Jurkat and HuT78 target cells and control K‐562 cells after 24 h of co‐culture (*n* = 3). Data are presented as mean ± SEM. ^****^ *p* < 0.0001 two‐way ANOVA. (N) The expression levels of CCR4 and CD7 in CD7 knockout (KO) CCRF‐CEM T‐lineage cells were examined by flow cytometry and compared with isotype controls. (O) Cytolytic activity of CD7N CAR‐T cells was evaluated against wild‐type and CD7 KO CCRF‐CEM cells after 24 h of co‐culture (*n*  =  3). Data are presented as mean ± SD. ns no significance, unpaired 2‐ tailed *t*‐test. (P) Cytolytic activity of CD7N CAR‐T cells against wildtype K‐562 and CCR4‐expressing K‐562 cells was assessed after 24 h of co‐culture (*n*  =  3). Results are shown as mean ± SEM. ^*^ *p* < 0.05, based on two‐way ANOVA.

To generate tandem CAR, we first compared the two configurations CCR4/CD7 vs. CD7/CCR4 using the same scFvs (Figure 3A). CCR4/CD7 tandem CAR exhibited stronger cytotoxic activity against Jurkat, ALL‐SIL, and CCRF‐CEM cells compared with the CD7/CCR4 CAR, whereas neither construct showed significant cytotoxicity toward the myeloid control cell line K‐562 (Figure 3B). Subsequently, we compared the cytotoxic effects of CD7 CAR‐T cells, CCR4 CAR‐T cells, and bispecific CCR4/CD7 CAR‐T cells generated from CD7‐negative T cells against the leukemia cell lines Jurkat, CCRF‐CEM, and ALL‐SIL. The results demonstrated that the CCR4/CD7 CAR‐T cells displayed stronger killing activity against Jurkat cells than either the CD7 or CCR4 CAR‐T cells alone (Figure 3C).

We then compared CD7N T cells and bulk T cells as immune effector cells for the CCR4/CD7 CAR. Transduction efficiency was detected by recombinant human CD7 protein (Figure 3D and Figure S1B). Of note, CAR expression was significantly lower in Bulk CAR‐T compared to CD7N CAR‐T (Figure 3D). The Bulk CAR‐T exhibited impaired expansion, compatible with fratricide, while the CD7N CAR‐T and NT Bulk showed good proliferation during the 8‐day culture ex vivo (Figure 3E,F). The cell compositions of CD7N CAR‐T and Bulk CAR‐T differed at the end of the culture period, with CD4/CD8 ratio higher in CD7N CAR‐T when compared with Bulk CAR‐T (Figure 3G). Furthermore, naïve (T_N_) T cells were enriched among Bulk CAR‐T, whereas central memory (T_CM_) T cells predominated among CD7N CAR‐T after transduction (Figure 3H; Figure S1C). Although LAG‐3 was more frequently expressed in CD7N CAR‐T cells, most of the cells (>70%) did not express any immune checkpoint receptors LAG‐3, PD‐1 or CTLA‐4 in either Bulk or CD7N CAR‐T group (Figure 3I). Compared to CD7N CAR‐T cells targeting CD7 or CCR4, fewer bispecific CD7N CAR‐T cells express LAG‐3 (Figure S1D). After 4 h and 24 h of co‐culture with Jurkat cells, Bulk and CD7N CAR‐T were assessed for degranulation and cytokine release ability, respectively. Both CAR‐T populations were capable of CD107 degranulation and cytokine release (Figure 3J–L). Furthermore, both CAR‐T products exhibited potent cytotoxicity even with E:T ratio <1:1 against all 4 T‐lineage cell lines, but not against the myeloid K‐562 control (Figure 3M).

The cytotoxic potency of the bispecific CD7N CAR‐T cells was further validated using a CD7‐negative relapse model. CD7N CAR‐T cells maintained robust killing activity against CD7‐knockout CCRF‐CEM cells or CCR4‐expressing K‐562 cells with low E:T ratio <1:1 (Figure 3N–P; Figure S1E). Collectively, these data suggested that the tandem CAR was effective and specific for a wide range of malignant T cells despite their variable levels of expression of CCR4 and CD7.

### 2.4 CCR4/CD7 Bispecific CD7N CAR‐T Demonstrated Antitumor Effect in Vivo

**FIGURE 4:**
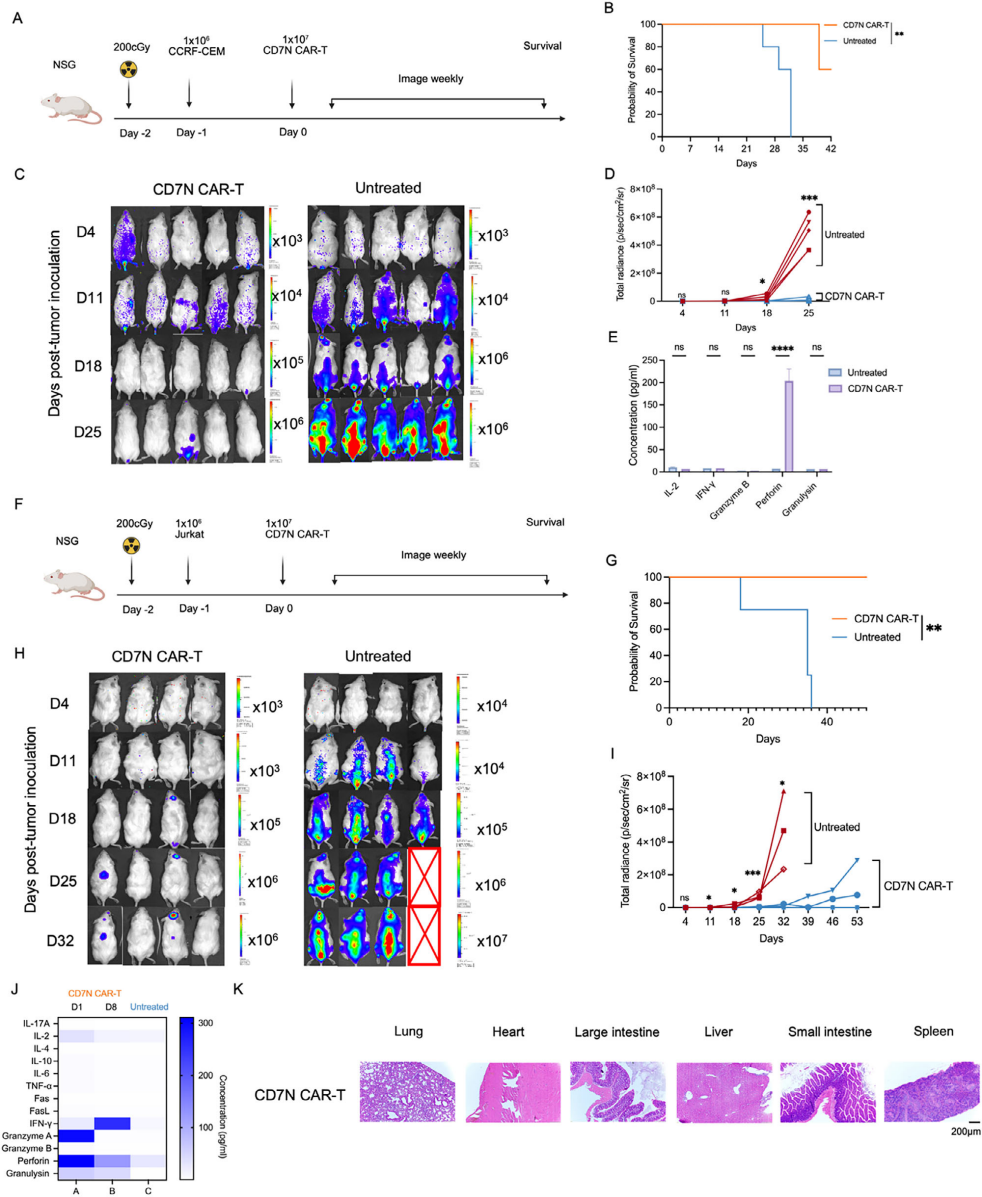
CCR4/CD7 bispecific CD7N CAR‐T Demonstrated Antitumor Effect in Vivo. (A) Diagram illustrating the experimental design. The 6–8 week old NSG mice were irradiated on day ‐2. On day ‐1, the mice were injected via tail vein with 1 × 10^6^ CCRF‐CEM‐firefly luciferase cells. (B) Survival curves of mice in the CD7N CAR‐T treated group and untreated group. ^**^ *p* < 0.01, log‐rank (Mantel‐Cox) test. (C) Bioluminescent imaging of tumor growth over time for CD7N CAR‐T treated groups and untreated group (n = 5 mice/per group). (D) Whole body tumor progression kinetics in each mouse, with each line corresponding to an individual animal. ns no significance, ^*^ *p* < 0.05, ^***^ *p* < 0.001 based on unpaired 2‐ tailed t‐test. (E) Cytokine levels detected in serum samples collected from mice tail vein on day 1 after CD7N CAR‐T cells injection (*n* = 2), compared with the untreated group. Data are presented as mean ± SEM. ns no significance, ^****^ *p* < 0.0001, two‐way ANOVA. (F) Diagram illustrating the experimental design of the second mouse model. The 6–8 week old NSG mice were irradiated on day ‐2. On day ‐1, the mice were injected via tail vein with 1 × 10^6^ Jurkat‐firefly luciferase cells. (G) Survival curve of mice in the CD7N CAR‐T‐treated group compared to the untreated group. ^**^ *p* < 0.01, log‐rank (Mantel‐Cox) test. (H) Bioluminescent monitoring of tumor growth over time for CCR4/CD7 bispecific CD7N CAR‐T treated group and untreated group (*n* = 4 mice/per group). (I) Whole body tumor progression kinetics, with each line denoting an individual animal, where significance is indicated by ns no significance, ^*^ *p* < 0.05, and ^***^ *p* < 0.001 based on unpaired 2‐ tailed t‐test. (J) Cytokine levels detected in serum samples collected from mice tail vein on day 1 and day 8 after CD7N CAR‐T cells injection (*n* = 2), compared with the untreated group. Data are presented as mean ± SEM. (K) Pathological examination of major organs (heart, large intestine, liver, lung, small intestine, and spleen) from a representative mouse in the CD7N CAR‐T‐treated group conducted using hematoxylin and eosin (H&E) staining, with magnification set at 200x.

The antitumor efficacy of CCR4/CD7 bispecific CD7N CAR‐T was assessed in vivo using a xenograft model. This model was established by injecting the CCRF‐CEM‐firefly luciferase cells into the tail vein of NSG mice (Figure 4A). Mice treated with CD7N CAR‐T cells exhibited a notable delay in tumor progression and a significant extension of survival when compared with untreated mice (Figure 4B,C,D). Mice treated with CD7N CAR‐T cells showed a significant increase of serum perforin levels on day 1 following CAR‐T administration (Figure 4E). This result substantiates the robust antitumor efficacy of CD7N CAR‐T cells in targeting CD7‐high CCR4‐high tumors in vivo.

To further evaluate the efficacy of the bispecific CD7N CAR‐T in vivo against another tumor cell line, we chose the Jurkat cell line, which was CD7+ but CCR4‐low/negative, mimicking the occasion of treatment of CCR4‐negative T‐cell malignancies or when CCR4 underwent antigen‐escape after targeted therapy (e.g., after CCR4 CAR‐T or mogamulizumab treatment) [30]. A second NSG mouse model was established by tail vein injection of Jurkat‐firefly luciferase cells (Figure 4F). In this model, the CD7N CAR‐T demonstrated significant antitumor activity even though the tumor cells expressed CD7 alone without CCR4 (Figure 4G–I). When cytokine and cytolytic molecules in the blood of the mice were measured on Day 1 and 8 after CAR‐T treatment (Figure 4J), high levels of Granzyme A and perforin on Day 1 and IFN‐γ on Day 8 were observed. Furthermore, pathological examinations of major organs revealed no microscopic abnormalities, indicating that CD7N CAR‐T did not induce histological toxicity in mice (Figure 4K). These results further validated the robust antitumor effects of bispecific CD7N CAR‐T cells against CD7highCCR4low tumors in vivo.

### 2.5 Enhancing the Safety of Bispecific CD7N CAR‐T Cells Through a EGFRt Switch

**FIGURE 5:**
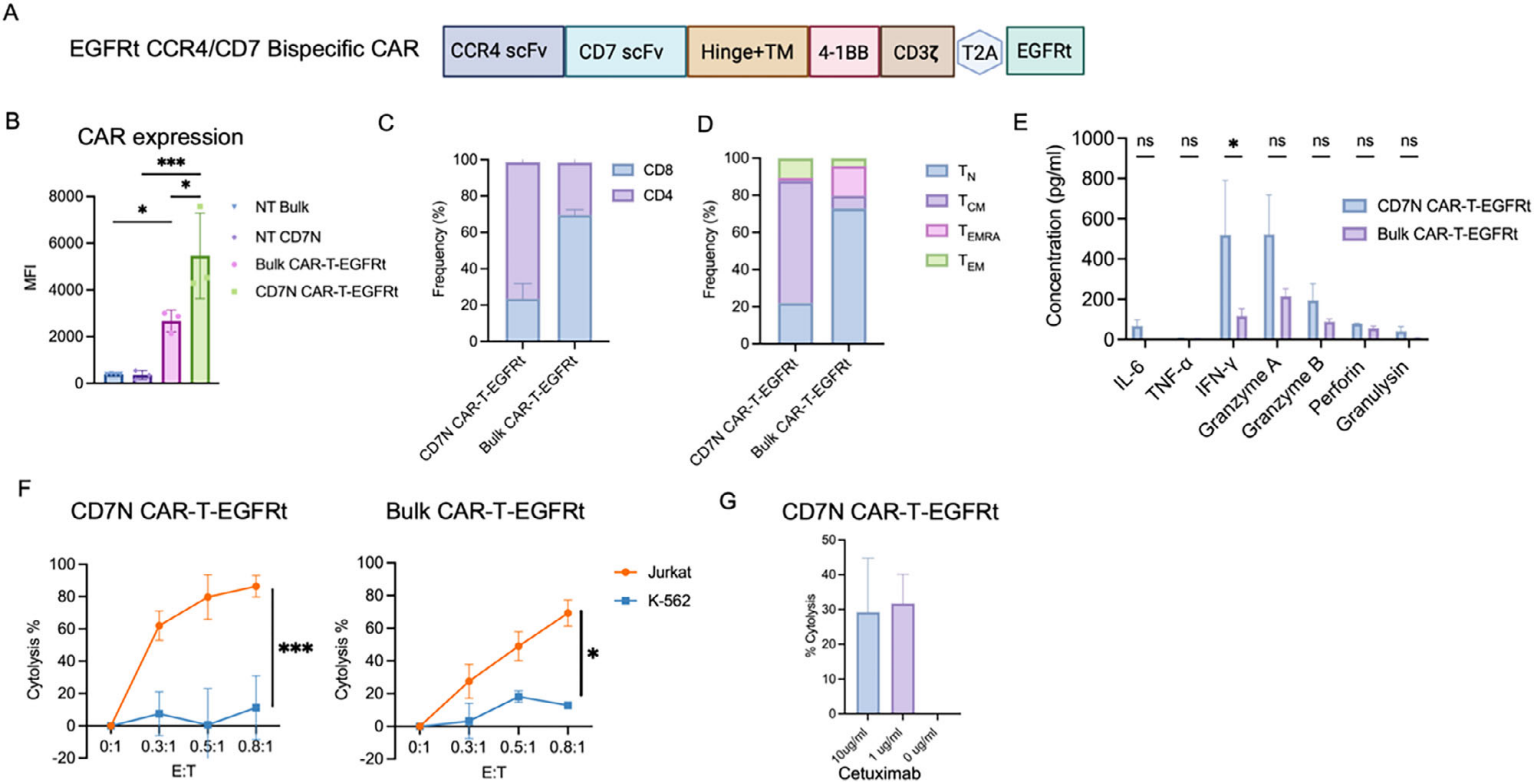
Incorporating an “off‐switch” into bispecific CD7N CAR‐T cells. (A) Schematic of the CCR4/CD7 bispecific CAR constructs with a EGFRt “off‐switch”. (B) Flow cytometry analysis of CAR expression on bispecific CCR4/CD7 CAR‐EGFRt‐transduced CD7N cells (CD7N CAR‐T‐EGFRt) and bulk T cells (Bulk CAR‐T‐EGFRt), compared with non‐transduced CD7N cells (NT CD7N) and bulk T cells (NT Bulk) (*n* = 3). Data are presented as mean ± SD, ^*^ *p* < 0.05, and ^***^ *p* < 0.001, one‐way ANOVA. (C,D) Analysis of CD4/CD8 ratio and memory phenotype of Bulk CAR‐T‐EGFR and CD7N CAR‐T‐EGFRt cell populations on day 10. Data are presented as mean ± SEM or average from three donors. (E) Cytokine release by Bulk CAR‐T‐EGFR and CD7N CAR‐T‐EGFRt after 24 h of co‐culture with Jurkat cells, as measured by LegendPlex assay (*n* = 4). Data are presented as mean ± SEM, ^*^ *p* < 0.05, two‐way ANOVA. (F) Cytolytic activity of CD7N CAR‐T‐EGFRt and Bulk CAR‐T‐EGFRt cells after co‐culture with Jurkat cells and control K‐562 cells for 24 h (*n* = 3). Data are presented as mean ± SEM, ^*^ *p* < 0.05, ^***^ *p* < 0.001, two‐way ANOVA. (G) Cytolytic activity against CD7N CAR‐T‐EGFRt cells under varying concentrations of Cetuximab (*n* = 3). NK cells from CD4+ T cell‐depleted PBMCs were utilized as effector cells. Data are presented as mean ± SEM.

Even though the CCR4/CD7 bispecific CD7N CAR‐T did not show any histologic abnormality or therapy‐related mortality in the mouse models, a safety switch is desirable in case of adverse events such as severe CRS, prolonged T‐cell aplasia, or secondary CAR+ T‐cell malignancies. To address these issues, we integrated a EGFRt domain into the CCR4/CD7 bispecific CAR construct with a bicistronic design (Figure 5A). This EGFRt system allowed selective elimination of the CAR‐T cells upon administration of the anti‐EGFR monoclonal antibody cetuximab. We first confirmed the expression of CCR4/CD7 bispecific CAR constructs, including Bulk CAR‐T‐EGFRt and CD7N CAR‐T EGFRt, using a recombinant human CD7 protein (Figure 5B). Furthermore, the addition of EGFRt did not alter the usual phenotype and cell composition of CD7N CAR‐T cells, including high CD4/CD8 ratio (Figure 5C) and predominant T_CM_ population (Figure 5D). The level of proinflammatory cytokine IFN‐γ produced by CD7N CAR‐T EGFRt was increased compared with Bulk CAR‐T‐EGFRt (Figure 5E). The EGFRt CAR‐T cells were able to effectively kill Jurkat cells while sparing K‐562 control cells (Figure 5F). The addition of cetuximab at concentrations as low as 1 µg/mL for 24 h led to considerable cytolysis of EGFRt CAR‐T cells (Figure 5G).

### 2.6 Enhancing CAR‐T Function Through c‐Jun Overexpression

**FIGURE 6:**
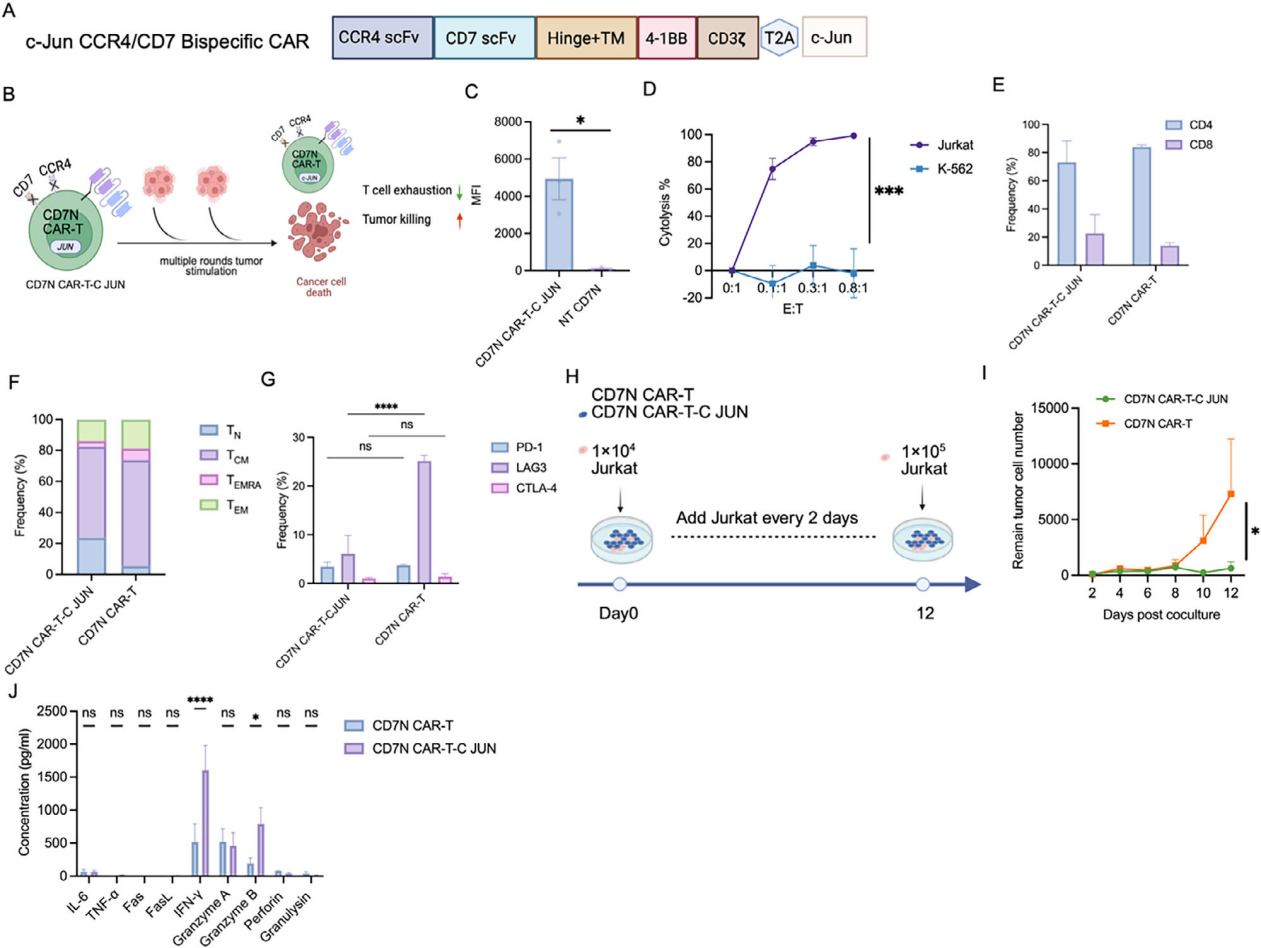
c‐Jun overexpression enhances the functional endurance of CAR‐T function. (A) Schematic of the CCR4/CD7 bispecific CAR vector encoding the transcription factor c‐Jun. (B) Illustration of c‐Jun overexpression leading to functional endurance of CCR4/CD7 bispecific CD7N CAR‐T cells (CD7N CAR‐T‐C‐JUN) against tumor cells. (C) Flow cytometry analysis of CAR expression on bispecific CD7N CAR‐T‐C‐JUN cells compared to non‐transduced CD7N cells (NT CD7N) (*n* = 3). Data are presented as mean ± SEM, ^*^ *p* < 0.05, unpaired 2‐ tailed *t*‐test. (D) Cytolytic activity of bispecific CD7N CAR‐T‐C‐JUN cells against Jurkat target cells (*n* = 3). Data are presented as mean ± SEM, ^***^ *p* < 0.001, two‐way ANOVA. (E,F) Analysis of the expression of CD4/CD8 ratio and memory phenotype of CD7N CAR‐T‐C JUN and CD7N CAR‐T cell populations on day 10. Data are presented as mean ± SEM or average from three donors. (G) Analysis of immune checkpoint receptor expression on bispecific CD7N CAR‐T‐C‐JUN and CD7N CAR‐T cell populations. Data are presented as mean ± SEM from three donors, ns no significance, ^*^^***^ *p* < 0.0001, two‐way ANOVA. (H,I) Schematic of the experiments with repeated tumor challenges for bispecific CD7N CAR‐T‐C‐JUN and CD7N CAR‐T cells, and the remaining tumor cell number after co‐culture (*n* = 2). Data are presented as mean ± SEM, ^*^ *p* < 0.05, two‐way ANOVA. (J) Cytokine release after 24 h of co‐culturing CD7N CAR‐T‐C‐JUN and CD7N CAR‐T cells with Jurkat cells. Jurkat cells co‐cultured with CD7N CAR‐T cells served as the control group. Measured by LegendPlex assay (*n* = 4). Data are presented as mean ± SEM, ns no significance, ^*^ *p* < 0.05, ^****^ *p* < 0.0001, two‐way ANOVA.

While safety is paramount, further clinical development of the CCR4/CD7 bispecific CAR will likely be more successful if the function of the CAR‐T cells without exhaustion could be improved. To enhance the functional endurance of the CD7N CAR‐T cells, we tested a CCR4/CD7 CAR constructs that overexpressed the transcription factor c‐Jun (Figure 6A,B). With this new vector, the expression of CAR and the cytotoxicity of the CD7N CAR‐T were tested in vitro (Figure 6C,D). The CD4/CD8 ratio was also unaltered (Figure 6E), and the T_CM_ population remained predominant (Figure 6F). Notably, without c‐Jun in the constructs, approximately 20–30% of the CAR‐T cells were positive for the exhaustion marker LAG3; however, with c‐Jun, the frequencies of LAG‐3+ CAR‐T cells significantly decreased to <10% (Figure 6G). Functionally, the CD7N CAR‐T‐C‐JUN cells exhibited enhanced endurance when challenged repeatedly with fresh Jurkat cells every two days in vitro, when compared to the CD7N CAR‐T without overexpression of c‐Jun (Figure 6H,I). These CD7N CAR‐T‐C‐JUN cells produced IFN‐γ and granzyme B rapidly within 24 h after co‐cultured with Jurkat cells (Figure 6J). Collectively, these findings demonstrated reduced expression of exhaustion marker and improved functional endurance in vitro when CD7N CAR‐T cells overexpressed c‐Jun.

### 2.7 Transcriptome Analysis of CCR4/CD7 Bispecific CD7N CAR‐T

**FIGURE 7:**
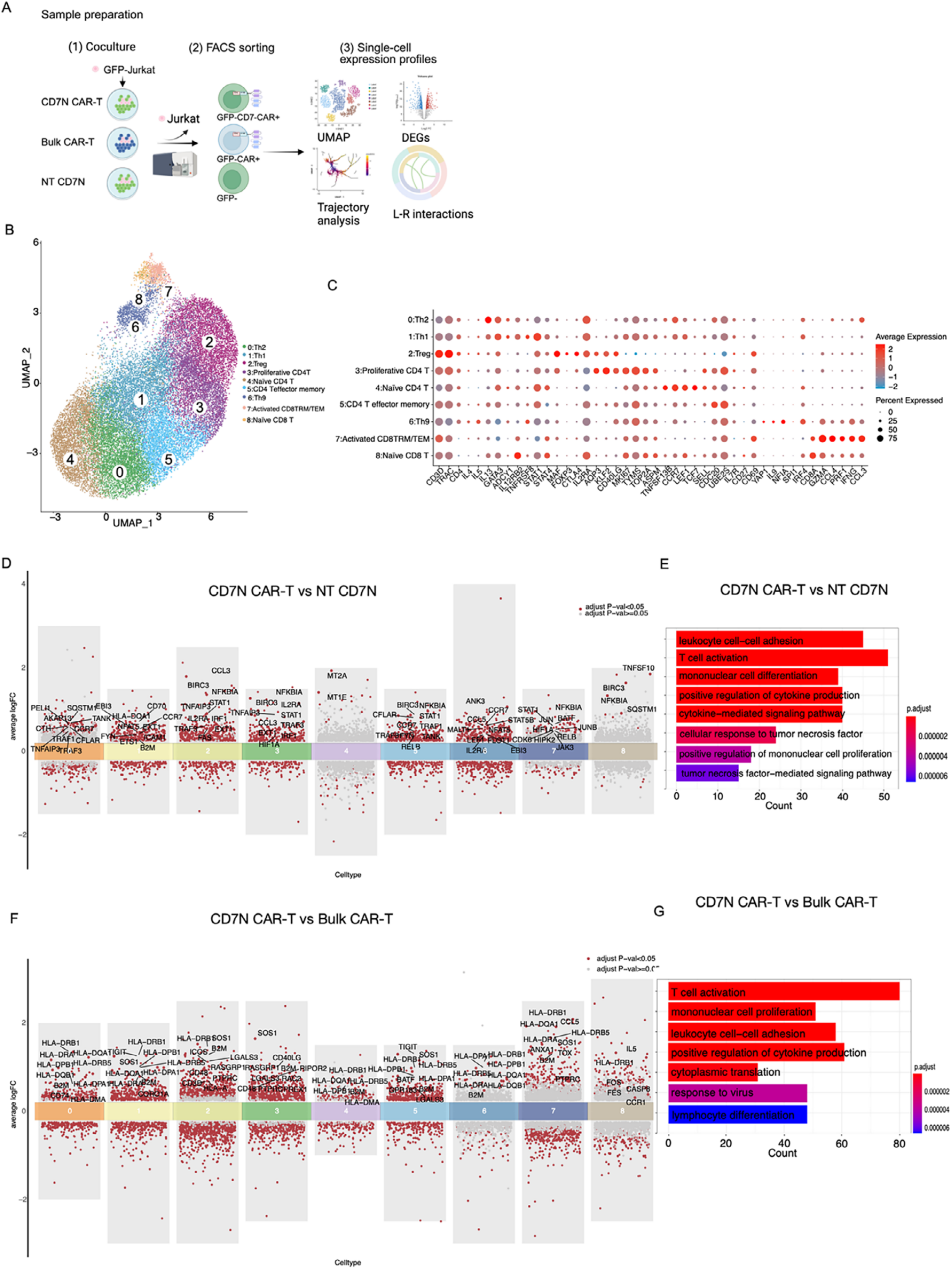
The transcriptome of bispecific CD7N CAR‐T cells. (A) Schematic representation illustrates the experimental design for transcriptome analysis among CD7N CAR‐T, Bulk CAR‐T, and NT CD7N. The cells were co‐cultured with GFP‐Jurkat cells at a 1:1 ratio for 24 h, followed by staining with specific antibodies and sorting via FACS to isolate GFP‐negative, CAR‐positive cells from the CD7N CAR‐T and Bulk CAR‐T groups, along with GFP‐negative NT CD7N cells. Sorted populations underwent scRNA‐seq and the data were compared by UMAP, DEGs, trajectory analysis and ligand‐receptor analysis to reveal gene expression differences among the three samples. (B) UMAP visualization of high‐quality single CAR‐T cells and T cells. Unsupervised clustering identified 9 clusters. (C) Dot plots of expression values for selected genes and proteins. Significant gene markers identified using Wilcoxon Rank Sum test. (D) Comparison of differentially expressed genes (DEGs) between CD7N CAR‐T and NT CD7N. Significant gene markers identified using Wilcoxon Rank Sum test. (E) Signaling pathways regulated by DEGs and their gene count in CD7N CAR‐T and NT CD7N.(F) DEGs between CD7N CAR‐T and Bulk CAR‐T. Significant gene markers identified using Wilcoxon Rank Sum test. (G) Signal pathways regulated by DEGs and their gene count in CD7N CAR‐T and Bulk CAR‐T.

In addition to c‐Jun, we sought to explore other molecular pathways which may further augment the function of CD7N CAR‐T. The experimental workflow is illustrated in Figure 7A. DEGs analysis among scRNA‐seq data revealed nine distinct T cell subsets within CD7N CAR‐T, Bulk CAR‐T and NT CD7N after stimulation with Jurkat cell line (Figure 7B,C). The CD8+ T cell clusters exhibited naïve (cluster 8) and activated tissue‐resident memory/effector memory (TRM/TEM) (cluster 7) subsets, with the latter characterized by high level of expression of granzyme A, IFN‐γ, CCL4, and CCL3. The CD4+T cell compartment comprised seven clusters, including IL13+ T helper 2 (Th2) cells (cluster 0); STAT1+ T helper 1 (Th1) cells (cluster 1); FOXP3+ regulatory T (Treg) cells (cluster 2); AQP3+ Ki‐67+ proliferative T cells (cluster 3); LEF1+ TCF7+ naive T (Tn) cells (cluster 4); CDC20+ effector memory T (Tem) cells (cluster 5); and IL‐9+ T helper 9 (Th9) cells (cluster 6). While Th1/Th2/naïve cells were relatively abundant in Bulk CAR‐T and Treg/AQP3+ were enriched in NT CD7N cells, the cluster distributions among CD7N CAR‐T appeared to be more balanced, with all clusters demonstrating an intermediate frequency distribution (Figure S1F,G).

When compared with NT CD7N cells, DEGs analysis followed by GO enrichment showed that clusters 1 (Th1) and 6 (Th9) of CD7N CART exhibited significantly higher expression of genes associated with cell‐cell adhesion pathways, including *ICAM1, FYN and ANK3*, (Figure 7D,E). *BIRC3*, which encodes cellular inhibitor of apoptosis protein 2 (cIAP2) and regulates tumor necrosis factor α (TNFα) activation of NF‐κB transcription factors [31, 32], was highly expressed in multiple clusters, including cluster 2, 3, 5, and 8 (Figure 7D), along with elevated expression of *TNFAIP3*, *TRAF3, NFKB1A* and *TNFSF10*. Collectively, these data suggest that the CAR engagement in CD7N cells when exposed to cancer cells resulted in a transcriptome profile characterized by T‐cell adhesion, activation, survival, and cytokine signalling particularly involving the tumor necrosis factor (TNF) superfamily.

Despite expression of the same CAR, the transcriptomes of CD7N CAR‐T cells and Bulk CAR‐T cells were significantly different, with CD7N CAR‐T showing remarkably higher levels of expression of *SOS1* in cluster 1, 3, 5, and 7; *PTPRC* in cluster 2, 3, and 7; and *LGALS3* in cluster 2, 3, and 5 (Figure 7F,G). While SOS1 is a key component of the Ras/MAPK pathway crucial for T‐cell proliferation and cytokine production [33], *PTPRC*‐encoded CD45 and *LGALS3*‐encoded galectin‐3 may signal through the Src and Bcl‐2 pathways to suppress apoptosis [34, 35]. Thus, when CD7N cells were used as immune effector cells instead of bulk T cells, the Src/Ras/Bcl‐2 pathways were more broadly activated at higher levels, particularly among (cluster 3) AQP3+ proliferative CD4+ T cells.

### 2.8 Evolution Trajectory and Receptor‐ligand Interactions of Cluster 3 AQP3+ Proliferative Cells

**FIGURE 8:**
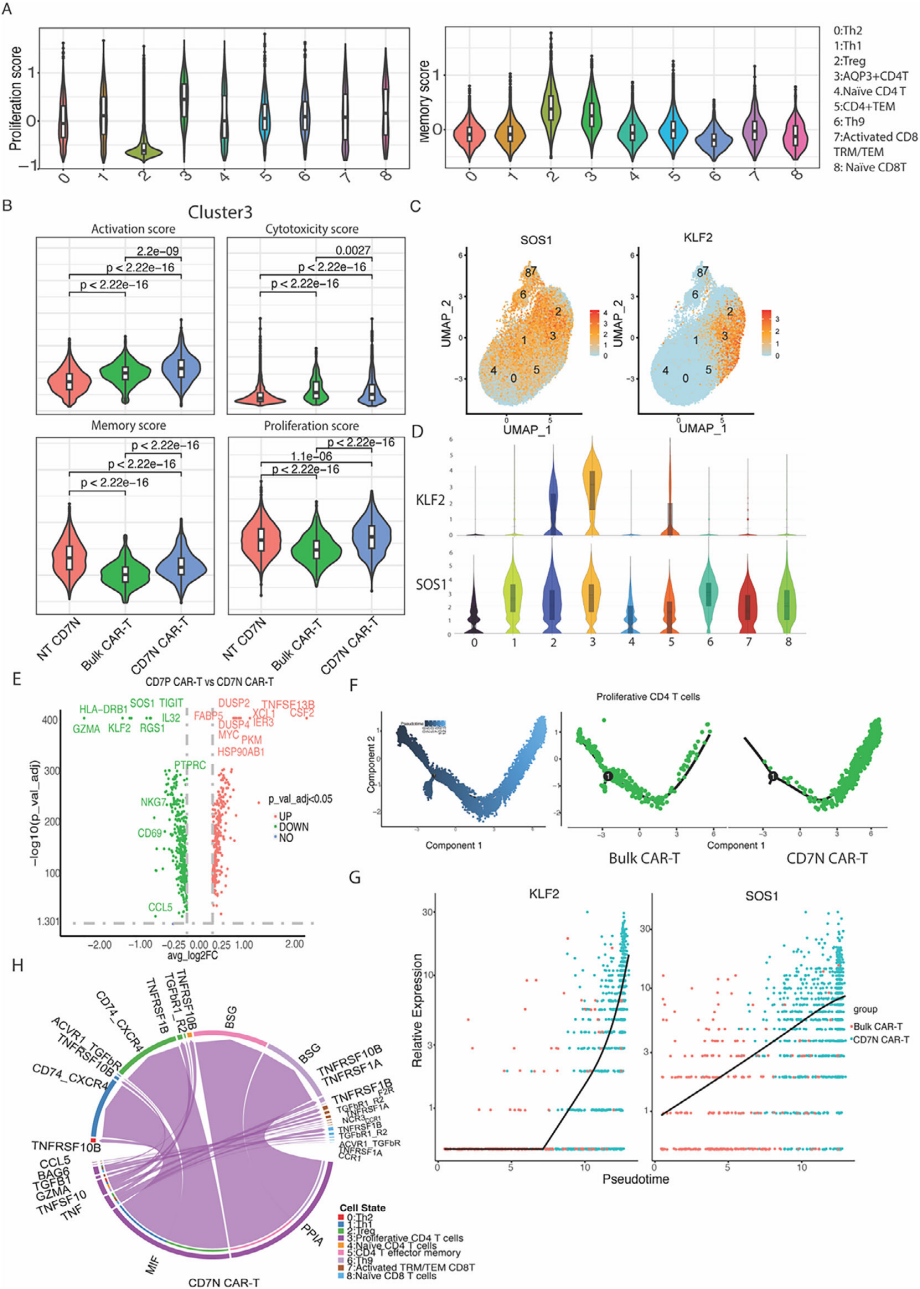
Trajectory analysis and cellular interactions of cluster 3 cells in CD7N CAR‐T. (A) Comparison of single‐cell expression profiles for proliferation and memory scores across clusters 0–8. Violin plots depict the distribution of representative gene expression within each cluster. (B) Single‐cell analysis comparing activation, cytotoxicity, memory, and proliferation scores in cluster 3 among CD7N CAR‐T, Bulk CAR‐T, and NT CD7N groups. Violin plots illustrate representative gene expression patterns, with significance assessed by the Wilcoxon Rank Sum test. (C) UMAP visualization demonstrating KLF2 and SOS1 expression levels within cluster 3 cells. (D) Violin plots illustrating the expression of KLF2 and SOS1 within cluster 0‐8. (E) Volcano plot showing DEGs between CD7‐positive (CD7P) CAR‐T and CD7N CAR‐T cells. Significant gene markers were identified using the Wilcoxon Rank Sum test. (F) The pseudotime evolution trajectories of CD7N CAR‐T, Bulk CAR‐T, and NT CD7N cells were reconstructed using Monocle2. Naïve CD4+ T cells were designated as the starting point of the trajectory. The progression of cluster 3 proliferative CD4+ T cells was analyzed separately within the CD7N CAR‐T and Bulk CAR‐T samples to trace their developmental process. (G) Relative expression levels of *SOS1* and *KLF2* and their pseudotime evolution trajectories in cluster 3 of the CD7N CAR‐T and Bulk CAR‐T groups. (H) L‐R interactions derived from cluster 3 were identified in CD7N CAR‐T group.

As cluster 3 AQP3+ Ki‐67+ CD4+ T cells were transcriptomically most distinct in the CD7N CAR‐T group compared with NT CD7N cells and Bulk CAR‐T cells, including signature genes such as *BIRC3*, *SOS1*, *PTPRC* and *LGALS3*, we further explored their biological properties. In comparison with the other eight clusters, cells in Cluster 3 exhibited elevated expression of genes related to cell proliferation (*TYMS, TOP2A, ASPM, VIM*, and *CD70*) as well as genes associated with memory formation (*CCR7, TCF7, IL7R, AQP3, CD27*, and *LTB*) (Figure 8A and Figure S2A,B). When compared to Bulk CAR‐T and NT CD7N, cluster 3 cells in CD7N CAR‐T had a transcriptome signature highest in expression of activation (*CD25, CD69*, and *SELL*) and cytotoxicity genes (*GZMA, GZMB, GZMH, GNLY, PRF1, NKG7* and *IFNG*) (Figure 8B). Moreover, proliferation genes were expressed at higher level in CD7N CAR‐T compared to Bulk CAR‐T, while memory phenotypes were less frequent in CD7N CAR‐T compared with NT CD7N cells. Thus, these data suggested that CAR‐signalling may drive proliferation and de‐differentiation of the transcriptome profile of CD7N CAR‐T. In addition to *SOS1* which was enriched in CD7N CAR‐T cells (Figure 7F), *KLF2* also displayed relatively high transcript level within cluster 3 (Figure 8C,D). When compared with their CD7‐positive counterparts, both *SOS1* and *KLF2* expression were significantly elevated in CD7N CAR‐T cells (Figure 8E). Trajectory analysis, using cluster 4 (naïve CD4+ T cells) as the root of the differentiation pathway (Figure 8F; Figure S2C,D), confirmed that cluster 3 cells from Bulk CAR‐T samples were predominantly localized near the root of the pseudotime trajectory, whereas in the CD7N CAR‐T samples, these cells were primarily located at the end of pseudotime, along with an enrichment of genes associated with mitosis and activation, as well as increased expression of *SOS1* and *KLF2* (Figure 8G; Figure S2E–H). Since KLF2 is associated with preserving stem‐like T cell characteristics and restraining terminal dysfunction, these findings suggest that bispecific CAR signalling may alleviate exhaustion‐related phenotypes in CD7N CAR‐T products [36], consistent with the phenotypes observed in the in vitro experiments (Figure S1D).

To investigate inter‐cluster communication by cluster 3 cells within the CD7N CAR‐T and Bulk CAR‐T populations, ligand‐receptor (L‐R) interaction analysis was performed. Compared to CD7N CAR‐T, the cluster 3 cells in Bulk CAR‐T had broader inter‐cluster communications and L‐R interactions (Figure S2I). In contrast, cluster 3 cells in CD7N CAR‐T interacted primarily with cluster 5 (CD4+Tem) and cluster 6 (Th9) via PPIA‐BSG, and with cluster 1 (Th1) and cluster 2 (Treg) via MIF‐CD74/CXCR4 (both of which could augment the activated ERK/MAPK and PI3K/AKT pathways previously demonstrated in CAR‐T cells with 4‐1BB costimulatory domain) [37, 38, 39]. Notably, cluster 3 cells engaged in broad L‐R interaction via the TNF superfamily with all other clusters except cluster 5 (Figure 8H). In contrast, Galectin‐9 (*LGALS9)* on cluster 3 of Bulk CAR‐T uniquely interacted with CD45 (*PTPRC*), CD44 and P4HB on multiple other clusters, which may contribute to inhibitory signalling among Bulk CAR‐T subsets [40, 41, 42].

## 3 Discussion

To overcome challenges such as fratricide, T‐cell exhaustion, antigen‐escape, and sustained T‐lymphopenia frequently observed with CAR‐T therapies for T‐cell malignancies, we first established a bispecific CAR‐T cell targeting CCR4 and CD7 using CD7‐negative cells. Incorporation of the c‐Jun transcription factor into the CAR construct confers resistance to exhaustion, while overexpression of EGFRt acts as a safety switch, enabling selective removal of CAR‐T cells if adverse events arise during their persistence. Although single‐target CD7 CARs and CCR4 CARs have been investigated in previous preclinical research [25, 26, 27], we first developed a bispecific CAR that simultaneously targets both CCR4 and CD7. CD7 has been the most common CAR target for immature T‐cell malignancies because of its high surface expression in >95% of T‐lymphoblastic leukemia/lymphoma and a subset of PTCL [43]. However, most mature T‐cell neoplasms are CD7 negative and has been used for diagnostic purpose. CCR4 was included in our bivalent CAR constructs to cover mature T‐cell malignancies and to mitigate the risk of CD7‐negative progressions such as those observed in CTCL over time [23, 24]. Prior preclinical studies have shown that CCR4 single‐targeting CAR was active against mature human T‐cell lymphoma [27]. In this study, the CCR4 and CD7 CARs were active for both single‐ and dual‐targeting in a collection of T‐cell malignancies with variable target expressions, including CTCL HuT78 which expressed only low levels of CD7 and CCR4. Other than T‐cell neoplasms, CD7 is also expressed in some myeloid malignancies such as 30% of acute myeloid leukemia and myelodysplastic syndrome, which was linked to poor prognosis [44]. Further attempts to apply this bispecific CAR to myeloid malignancies are worth investigating.

Fratricide‐resistant CD7 CAR‐T has been developed using methods such as genome editing, surface downregulation, transient CAR‐signalling inhibition, or use of CD7 negative effector cells [6, 8, 25, 45, 46, 47]. One of the major concerns of using CCR4/CD7 bispecific CAR was whether it may further exacerbate the fratricide of CAR‐T cells, resulting in manufacturing and treatment failure, as only a small fraction of blood T cells is CCR4/CD7 double negative and CD7 gene‐editing/surface downregulation alone cannot avoid CCR4‐mediated CAR‐T fratricide. Furthermore, CD7 knockout using CRISPR/Cas9 approach is limited by unintended genetic alterations, gene‐editing mosaicism, regulatory hurdles, manufacturing complexity and cost. In contrast, our CD7N selection process is non‐genotoxic and >95% purity of CD7‐negative cells could be expediently attained after a simple CD7‐depletion procedure that takes only two hours. The CD7N cells proliferated well in a culture system with IL‐7 and IL‐15, achieving >10‐fold expansion in 8 days after bivalent CAR vector transduction with minimal fratricide compared with unselected starting cells (Figure 3E). Prior analysis of leukapheresis products as starting material from adult lymphoma patients reported a median of 2.55 × 10^9^ CD3+ lymphocytes [48]. In our study, CD7‐ CCR4‐ cells comprised a median of 2.9% of CD3+ cells, yielding approximately 70 million starting cells for CD7N CAR‐T manufacturing. With >50% transduction efficiency consistently observed with our bispecific CAR vectors and 10 to 20‐fold expansion within 8 days post‐induction (Figure 3E,F; Figure S1B), the final yield would exceed 350 million CAR+ cells, corresponding to >5 million cells/kg for a 70‐kg adult (well within the dosing range of FDA‐approved CAR‐T products).

Adverse events are common after CAR‐T therapy; some could be life‐threatening, including severe CRS, T‐lymphopenia, or second malignancies. While complete T‐cell aplasia was not observed in most recipients of CD7 CAR‐T because of recovery of naturally CD7negative T cells, the use of CCR4/CD7 bispecific CAR may potentially exacerbate the risk of immunodeficiency. Low level of CCR4 may be expressed in organs such as lungs resulting in on‐target off‐tumor adverse events. In this regard, our CCR4 CAR‐T cells were cytotoxic to CCR4+ T cells obtained from healthy mice (Figure S1H); however, no lung toxicity was observed in the mouse models. With CD19 CAR‐T, previous studies have demonstrated that some relapses were attributable to epitope‐masking, when the CAR gene was unintentionally introduced into a leukemic B cell during T cell manufacturing, resulting in cis interaction of the CAR to the CD19 epitope on the surface of leukemic cells. For T‐lineage leukemia, this adverse event is not readily preventable by T‐cell enrichment strategy before transduction as the leukemia cells may express the same T‐cell antigens. Overexpression of c‐Jun in CAR‐T cells may further increase the risk of leukemia proliferation. Given these limitations, we tested the feasibility of elimination of the CAR‐T cells in case such an adverse event occurs by incorporating EGFRt in the bispecific CAR vector design. While no alteration in phenotype and function of the CCR4/CD7 CAR‐T was observed, the cells could be readily lysed by a commercially available antibody cetuximab at a concentration as low as 1 µg/mL (Figure 5G), markedly lower than the usual trough concentration (49 µg/mL) in the plasma of cetuximab recipients [49]. Rare cases of severe lung toxicities have been reported after cetuximab treatment [50], with a median onset of about 100 days after repeated weekly doses [51]. For our bispecific CAR‐T cells, we found that an exposure to cetuximab at low concentrations for as brief as 24 h could lead to cytolysis of one‐third of the CAR‐T cells. Thus, the relatively short duration of cetuximab exposure required for CAR‐T elimination might be less toxic.

Given that CAR‐T can be turned off with safety switch, we sought to determine whether turning on proto‐oncogenes constitutively can reduce CAR‐T exhaustion, another common mechanism of cancer recurrence. In this regard, c‐Jun and basic leucine zipper ATF‐like transcription factor (BATF) may induce exhaustion‐resistance by displacing other inhibitory family members of the activator protein 1 (AP‐1) and interferon regulatory factor (IRF) complexes, which normally drive T cells toward exhaustion and terminal differentiation [52, 53]. Previously, overexpression of c‐Jun in Bulk CAR‐T cells has been shown to improve anti‐tumor potency and promote cell survival [54]; however, its effects on CD7N cells are uncertain, as CD7‐negative T cells represent a more terminally differentiated population and exhibit reduced activation and proliferation potential when compared to CD7‐positive T cells [55]. Indeed, we demonstrated herein that CCR4/CD7 bispecific CD7N CAR‐T exhibited functional exhaustion after repeated challenges with leukemia cells in vitro; however, overexpression of c‐Jun within these CAR‐T cells improved their functional endurance (Figure 6I).

Single‐cell transcriptomic analysis revealed that CD7N CAR‐T cells were predominantly composed of CD4+ transcriptomic changes upon tumor exposure. Significant alterations of gene expression were observed in pathways governing cell adhesion, activation, survival, and cytokine signalling. A unique subset of AQP3+ cells was particularly notable, which had a transcriptome signature of broadly activated Src/Ras/ERK/Bcl‐2 pathways, with inter‐cluster ligand‐receptor interactions involving PPIA/MIF and the TNF superfamily. *SOS1* and *KLF2* were identified as the most prominently and differentially upregulated genes in CD7N CAR‐T cells (Figure 8C,D,E,G). SOS1 regulates PIK3CD, of which gain‐of‐function mutations have been shown to enhance CAR‐T signalling, cytokine production, and leukemia cell killing [56]. Recently, another study demonstrated that KLF2 can promote the differentiation of effector CAR‐T cells and prevent terminal exhaustion, thereby enhancing CAR‐T efficacy [57]. Collectively, these data raise the possibility that modulation of *SOS1* and *KLF2* may represent a potential avenue for enhancing CAR‐T cell efficacy.

While the current study demonstrated the efficacy and safety of CD7N bispecific CAR‐T cells in vitro and in vivo using the xenograft model, several limitations must be acknowledged. Before clinical translation, further validation in models such as patient‐derived xenografts is warranted. Although c‐Jun overexpression was observed in this study to maintain the functional endurance of CAR‐T cells and reduce exhaustion after repeated tumor challenges in vitro, whether it can sustain CAR‐T cell persistence in vivo remains to be determined. Future in vivo studies using CAR constructs incorporating both c‐Jun and EGFRt will be required to conclusively evaluate long‐term functional persistence, late adverse events, and the permanence of cetuximab‐mediated safety switch. While our L‐R interaction analyses uncovered the involvement of PPIA/MIF in inter‐cluster CAR‐T communication after exposure to cancer cells, most relevant L‐R interactions would be with cells outside of the T‐cell compartment, underscoring the importance of broader interrogation in future analyses. Although alterations in *SOS1* and *KLF2* transcription signatures were observed in CD7N CAR‐T cells, future elucidation of related molecular mechanisms underlying CAR‐T cell enhancement is required. Additionally, the generalizability of our findings to other non–T‐lineage malignancies, including myeloid neoplasms, warrants further investigation.

In summary, this study has provided a systemic characterization of CCR4/CD7 bispecific CAR‐T engineered from CD7N T cells, delineating their rational design framework, manufacturing feasibility, functional potency and phenotypic stability. The demonstrated resistance to both fratricidal elimination and CAR‐T exhaustion, coupled with robust activity against antigen‐heterogenous tumors, established a compelling preclinical foundation for further clinical translation.

## 4 Materials and Methods

### 4.1 Generation of CCR4/CD7 CAR Constructs

The two scFv CCR4‐CAR (patent: US20210315985A1) and anti‐CD7 scFv sequences (patent: US10550183) were linked by a flexible (GGGGS) × 5 linker and then fused to the sequence containing a CD8 α hinge, a transmembrane domain, a 4‐1BB (CD127) co‐stimulatory domain, and intracellular CD3ζ signal domain. The CCR4/CD7 CAR fragment was cloned into the lentiviral vector with human elongation factor 1 alpha (Ef1α) promoter (pLenti‐EF1a‐Backbone). The synthesized construct was created by commercial gene synthesis (Genewiz company, USA). The truncated epidermal growth factor receptor (EGFRt), as described in patent US10189903B2, was linked to CAR fragments via a T2A sequence and functioned as a suicide gene, allowing for the elimination of CAR‐T cells through the use of biotinylated Erbitux (Cetuximab) antibody. Detection of EGFRt was carried out using the anti‐EGFRt antibody (R&D Systems, cat. # FAB9577B). Furthermore, the *JUN* gene, derived from the human A549 cDNA library and corresponding to the NCBI accession number NM_002228.4, was incorporated into the CCR4‐CD7‐CAR construct through a T2A sequence.

### 4.2 CD7‐Negative T Cell Isolation

To isolate T cells and CD7‐negative T cells, PBMCs were isolated from healthy donor buffy coats provided by the Hong Kong Red Cross. The separation was performed with Lymphoprep (STEMCELL Technologies; Cat. # 07811), following the protocol supplied by the manufacturer. On Day 0, before CD7 depletion, the T cell was first selected from human PBMCs via pan‐T selection kit (Miltenyi Biotec; Cat. # 130‐096‐535) via magnetic bead separation using the Miltenyi Biotec magnetic separator (midi separator Miltenyi Biotec; Cat. #120‐122‐562). The CD7‐negative T cells were then selected via CD7 depletion using the biotin‐conjugated anti‐CD7 antibody (Miltenyi Biotec; Cat. #130‐123‐562) and anti‐biotin magnetic beads (Miltenyi Biotec; Cat. #130‐090‐485). The cell suspension was loaded onto an LS column (Miltenyi Biotec; Cat. #120‐122‐562) and separated using the magnetic separator as we did before [58].

### 4.3 Cell Culture

The isolated CD7‐negative T cells and bulk T cells were cultured in TexMACS Medium (Miltenyi Biotec; Cat. #130‐097‐196) supplemented with human interleukin‐2 (IL‐2; Miltenyi Biotec; 10 ng/mL) on days 0–3. Subsequently, the medium was changed to TexMACS Medium containing human interleukin‐7 (IL‐7; Peprotech; 10 ng/mL) and human interleukin‐15 (IL‐15; Peprotech; 10 ng/mL) for the following days. The Jurkat, ALL‐SIL, CCRF‐CEM, and K‐562 cell lines were cultured in RPMI medium supplemented with 10% fetal bovine serum (FBS; Thermo Fisher Scientific, Waltham, MA, USA) and 1% penicillin‐streptomycin (Thermo Fisher Scientific, Waltham, MA, USA) as we described before [59]. HuT78 cells were cultured in Iscove's Modified Dulbecco's Medium (IMDM) with 10% FBS and 1% penicillin‐streptomycin (Thermo Fisher Scientific).

### 4.4 Generation of Bispecific CD7N CAR‐T

The isolated CD7‐negative T cells were activated with T Cell TransAct (Cat. #130‐128‐758) at a 1:100 ratio for 24 h on day0. RetroNectin (Takara, Japan) were coated at 20 µg/mL in the plate at 4°C overnight. On day 1, the activated CD7‐negative T cells were transduced with a lentivirus at a multiplicity of infection (MOI) of 30 for 48 h. The in vitro and in vivo experiments were performed on days 7–12 using the transduced CD7 negative‐T cells.

### 4.5 Flow Cytometry

PE‐conjugated mouse anti‐human CD7 antibody (clone:CD7‐6B7;Biolegend, USA) and APC‐conjugated CCR4 mouse anti‐human CD7 antibody (clone: L291H4;Biolegend, USA) were used to detect the surface expression of CD7 and CCR4. PE Mouse IgG2a, κ Isotype Ctrl Antibody and APC Mouse IgG1, κ Isotype Ctrl Antibody were used as isotype control. The expression of CAR was detected using biotinylated goat anti‐mouse IgG (H+L) secondary antibody (Catalog No. 31800, ThermoFisher, USA), FITC‐conjugated anti‐biotin (Miltenyi Biotec, Germany), APC streptavidin (Biolegend, USA), PE‐conjugated Protein‐L, anti‐EGFRt antibody (R&D Systems, USA) and the APC‐Labeled Human CD7 Protein (ACRO Biosystems, USA). Live and dead cells were distinguished using 7‐amino‐actinomycin D (7‐AAD) viability staining solution (Biolegend, USA). PE conjugated anti‐human CD4 (clone: SK3) and APC conjugated anti‐human CD8 (clone: SK1), PE anti‐human CD279 (PD‐1) (clone: A17188A; Biolegend, USA), Brilliant Violet 785 anti‐human CD223 (LAG‐3) (clone: 11C3C65), PE anti‐human CD25 (clone: M‐A251), APC anti‐human CD69 (clone: FN50), Brilliant Violet 785 anti‐human CD152 (CTLA‐ 4) (clone: BNI3), PE anti‐human CD45RA (clone: HI100), APC anti‐human CD197 (CCR7) (clone: G043H7) were used to detect the activation and differential phenotype, exhaustion marker of bispecific CD7N CAR‐T and Bulk CAR‐T cells. Degranulation of bispecific CD7N CAR‐T and Bulk CAR‐T cells was detected by Pacific Blue anti‐human CD107a (LAMP‐1) (clone: H4A3) after co‐culturing with Jurkat cells for 4 h and detected by flow cytometry as we did before [60, 61].

#### 4.5.1 Cytokine Measurement

The supernatant was collected by 24‐h coculture of CD7N CAR‐T and target cells. The cytokine release level including IL‐2, IL‐4, IL‐6, IL‐10, IL‐17A, IFN‐γ, TNF‐α, soluble Fas, soluble FasL, Granzyme A, Granzyme B, Perforin, and Granulysin of the CAR‐T cells was detected by LEGENDplex Human CD8/NK Panel (13‐plex) (Biolegend, USA) using flow cytometry [62].

#### 4.5.2 Cytolysis Assay

##### 4.5.2.1 CFSE‐Based Cytolysis Assay

To determine the cytolysis of CD7N CAR‐T, the target cells CCRF‐CEM, ALL‐SIL, Jurkat, HuT78 and K‐562 were stained with 0.5 µM CFSE for 15 min at room temperature, and cocultured with CD7N CAR‐T at E:T ratios of 0:1, 0.1:1, 0.3:1, 0.8:1 for 24 h. After 24 h of coculture, the plate was centrifuged at 300 ×g for 5 min and the supernatant was collected for subsequent cytokine level detection. The cell was resuspended in 200 µL PBS + 0.5%BSA which added 20 µL Count Bright Absolute Counting Beads (ThermoFisher; Cat.log C36950). The remaining live CFSE‐positive tumor cells were detected by flow cytometry.

##### 4.5.2.2 Fluorescence Signal‐Based Cytolysis Assay

The target cells CCRF‐CEM, ALL‐SIL, Jurkat, HuT78 and K‐562 were expressed mCherry signal and incubated with effector bispecific CD7N CAR‐T and Bulk CAR‐T cells in 96 well plates for 24 h at E:T ratios at 0:1, 0.1:1, 0.3:1, 0.8:1 for 24 h. The remaining live mCherry‐positive tumor cells were detected by flow cytometry.

### 4.6 Repeat Antigen Stimulation Assay

On day 0, 8000 CD7N bispecific CAR‐T and bispecific CD7N CAR‐T ‐C JUN were cocultured with the Jurkat expressing GFP at an E:T ratios of 0.8:1, and 1×10^5^ Jurkat‐GFP was added into the plates every 2 days. The remaining live GFP+ Jurkat cells were determined by flow cytometry every two days.

### 4.7 Cetuximab‐mediated Depletion of EGFRt‐expressing CD7N CAR‐T Cells

The PBMCs were incubated with CD4 microbeads (Miltenyi Biotec, Germany) for 15 min at 4°C. The CD4 negative PBMCs were selected by the LS column (Miltenyi Biotec, Germany). The cells were used as effector cells and cocultured with the CAR‐T cells at 0, 1 and 10 µg/mL cetuximab for 24 h, the remaining live CAR‐positive T cells were detected by flow cytometry.

### 4.8 Mouse Xenograft Models

The study utilized 6–8‐week‐old male NOD.Cg‐Prkdcscid Il2rgtm1Wjl/SzJ (NSG) mice, originally obtained from The Jackson Laboratory in the United States. The mice were bred under an AAALAC International accredited program at the Centre for Comparative Medicine Research, HKU, maintained under Specific Pathogen Free (SPF) conditions. All research procedures were reviewed and approved by the HKU Committee on the Use of Live Animals in Teaching and Research (CULATR# 23–266) and conducted under license from the Hong Kong SAR Government's Department of Health.

Prior to experimentation, the 6–8 week‐old NSG mice were gamma irradiated at a dose of 200cGy on day ‐2. On the day ‐1, mice were administered an intravenous tail vein injection of 1 × 10^6^ Jurkat‐firefly luciferase or CCRF‐CEM‐firefly luciferase cells. Subsequently, they were randomly assigned to various treatment groups, followed by the administration of 1 × 10^7^ CD7N CAR‐T cells on day 0. Tumor burden was monitored through intraperitoneal injection of D‐Luciferin potassium salt (GoldBio, Cat. # LUCK‐100) and IVIS imaging at designated time points. Living Image software (PerkinElmer, USA) was used for luminescence visualization and quantification.

After tumor implantation, mice were randomly assigned to receive CD7N CAR‐T treatment or no treatment. Mice were euthanized based on observed symptoms of distress, including hunched posture, significant weight loss, mobility issues, paralysis, or luminescence exceeding 1 × 10^8^. Organs were harvested and submitted for HKU pathology assessment, including hematoxylin and eosin (H&E) staining. Blood samples were collected via the tail vein, and the serum was analyzed using the LEGENDplex Human CD8/NK Panel (13‐plex) (Biolegend, USA) on the online platform (https://legendplex.qognit.com).

### 4.9 Single‐ Cell RNA Sequencing

#### 4.9.1 Sample Preparation

CD7N CAR‐T, Bulk CAR‐T, and NT CD7N cocultured with GFP‐Jurkat cells at 1:1 for 24 h. The above population were stained by APC‐human CD7 protein (ACRO Biosystems, Newark, Delaware, USA), PE‐conjugated mouse anti‐human CD7 antibody (clone:CD7‐6B7;Biolegend, USA) and 7‐AAD Viability Staining Solution (Biolegend, USA) were used to sort GFP‐negative, CD7‐negative, APC‐positive and 7‐AAD‐negative cells in the CD7N CAR‐T group by Fluorescence‐Activated Cell Sorting (FACS). FACS sorting was performed using FACSAria Fusion Cell Sorter (BD Biosciences, Franklin Lakes, New Jersey, USA).

#### 4.9.2 Single‐Cell RNA Sequencing Preparation and Sequencing

Single‐cell encapsulation and cDNA libraries were performed using the Chromium Next GEM Single Cell 3′ Reagent Kits v3.1 (10x GENOMICS). The single‐cell RNA sequencing (scRNA‐seq) library was constructed using the cell suspensions were loaded onto the Chromium Controller instrument to generate single‐cell Gel Beads‐In‐Emulsions (GEMs), where individual cells were isolated into droplets along with gel beads coated with unique primers bearing 10x cell barcodes, unique molecular identifiers (UMIs), and poly(dT) sequences. The GEM‐reverse transcriptions were then performed, followed by cDNA library amplification, fragmentation, end repair, A‐tailing, adaptor ligation, and sample index PCR. The final libraries were sequenced using the NovaSeq 6000 platform (Illumina) to a depth of approximately 500 million reads per library with 2×150 read length.

#### 4.9.3 Quality Control

The raw scRNA‐seq data were pre‐processed using the cellranger count (v7.1.0) pipeline. Cells with fewer than 500 features or more than 7000 features detected, as well as those with a mitochondrial gene content greater than 15%, were filtered out as low‐quality cells. Additionally, potential doublets were identified and excluded using the DoubletFinder R package (v2.0.3). The quality‐controlled and normalized data were further analyzed using the Seurat R package (v4.3.0).

#### 4.9.4 Cell Clustering and Cell‐type Annotation

To identify distinct cell clusters, the top 2000 highly variable genes (HVGs) were selected, and principal component analysis (PCA) was performed. Batch effects were corrected using the RunHarmony function in Seurat. Knn graphs were constructed based on the Euclidean distance in the 50D PC space, and the Louvain‐Jaccard graph‐based method was used to identify the main cell clusters. The clusters were then visualized using Uniform Manifold Approximation and Projection (UMAP). Differential gene expression analysis was conducted using the Seurat FindAllMarkers function, and cell types were manually annotated based on the cluster‐specific marker genes.

#### 4.9.5 Differentially Expressed Genes (DEGs) Analysis

For further analysis of T cells, the same preprocessing and clustering steps were repeated. Subclusters were identified, and their marker genes were determined using the Seurat FindAllMarkers function. Differential gene expression analysis was performed between relevant groups within each subcluster, and the significance of differences was assessed using the Wilcoxon rank‐sum test with Bonferroni correction.

#### 4.9.6 Gene Ontology (GO) Enrichment Analysis

GO enrichment analysis was conducted using the clusterProfiler R package (v4.2.2) to identify biological processes associated with the DEGs in each subcluster. The significance of enrichment was determined using a hypergeometric test, and the Benjamini‐Hochberg method was used to control the false discovery rate (FDR).

#### 4.9.7 Cell‐Cell Ligand‐receptor Interaction Analysis

Cell‐cell communication analysis was performed using the CellChat R package (v2.1.2). Differential cell‐cell interaction networks were reconstructed to identify the changes in ligand‐receptor interactions between different cell types or conditions.

#### 4.9.8 Trajectory Analysis

The trajectory analysis was performed using the Monocle2 R package (v2.22.0) to reveal cell state transitions in CD4+T cells among CD7N CAR‐T, Bulk CAR‐T, and NT CD7N. We used the DEGs of each subcluster in the intended cell types identified through Seurat FindAllMarkers function with the default parameters, to sort the cells in pseudotime order. Dimensional reduction and cell ordering were performed using the DDRTree method and the orderCells function. Lastly, plot_cell_trajectory function was run for visualization. The DEGs changes along the pseudo‐time were also determined with the differentialGeneTest function with default parameters, and the DEGs with the adjusted *p* value (qval <0.0001) were visualized with the plot_pseudotime_heatmap function.

### 4.10 Graphs and Statistical Analysis

Graphs and statistical analyses were performed using GraphPad Prism Software, version 9. All results are based on a minimum of three independent experiments. Data are presented as the mean ± standard error (SE) or mean ± standard deviation (SD). Statistical significance was determined using one‐way analysis of variance (ANOVA), two‐way ANOVA, Student's t‐test, or the log‐rank test, depending on the specific analysis. P‐values are reported as follows: not significant (ns), ^*^ *p* < 0.05, ^**^ *p* < 0.01, ^***^ *p* < 0.001, and ^****^ *p* < 0.0001.

## Author Contributions

Conceptualization: W.L., S.L. . Data curation: S.L., Y.L., A.R. . Funding acquisition: W.T., W.L. . Methodology: S.L., Y.L., A.R., H.T., S.C., M.H., W.C., K.L., Y.L. . Project administration: W.T., W.L. . Writing – original draft: S.L., W.T., W.L. . Writing – review&editing: S.L., Y.L., A.R., H.T., W.T., W.L. . All authors have reviewed and commented on the submitted manuscript.

## Ethics Statement

The mice were maintained and bred in a SPF environment at the AAALAC International‐accredited Centre for Comparative Medicine Research, HKU. All procedures received approval from the HKU Committee on the Use of Live Animals in Teaching and Research (CULATR# 23–266) and performed under a license issued by the Department of Health, Hong Kong SAR Government.

## Conflicts of Interest

The authors declare no conflicts of interest.

## Declaration of Generative AI and AI‐Assisted Technologies in the Writing Process

During the writing process, the author utilised ChatGPT to proofread the language, ensuring clarity and grammatical accuracy. Following the use of this tool, the author reviewed and edited the content as necessary and assumes full responsibility for the publication's content.

## Supporting information




**Supporting File**: advs74395‐sup‐0001‐SuppMat.docx.

## Data Availability

The data that supports the findings of this study are available from the corresponding authors, W.T and W.L, upon reasonable request.
