# Supplementary material for: Generation of CCR4/CD7 Bispecific CAR‐T Cells Resistant to Fratricide and Exhaustion
Source: Adv Sci (Weinh). 2026 Feb 26;13(35):e21443. doi: 10.1002/advs.202521443 (PMC13292163; doi:10.1002/advs.202521443)
Supplement: Supplementary file 1 — Supporting File: advs74395‐sup‐0001‐SuppMat.docx. [file ADVS-13-e21443-s001.docx]

**Generation of CCR4/CD7 bispecific CAR-T cells resistant to fratricide and exhaustion**

**Sile Li^1^, Yuanxin Li^1^, Asif Rashid^1^, Hong Kee Tan^1^, Shing Chan^1^, Man Yan Hui^1^, Wilson Yau Ki Chan^2^, Kee See Lam^2^, Yinping Liu^1^, Wenwei Tu^1^, Wing Leung^1,3^**

**Correspondence:** Wenwei Tu; [wwtu@hku.hk](mailto:wwtu@hku.hk), Wing Leung; [leungwhf@hku.hk](mailto:leungwhf@hku.hk)

1. Department of Paediatrics and Adolescent Medicine, Li Ka Shing Faculty of Medicine, The University of Hong Kong, Hong Kong SAR, PR China
2. Department of Paediatrics and Adolescent Medicine, Hong Kong Children's Hospital, Hong Kong SAR, PR China
3. Children’s Blood and Cancer Centre, KK Women’s and Children’s Hospital, SingHealth Duke-NUS, Singapore


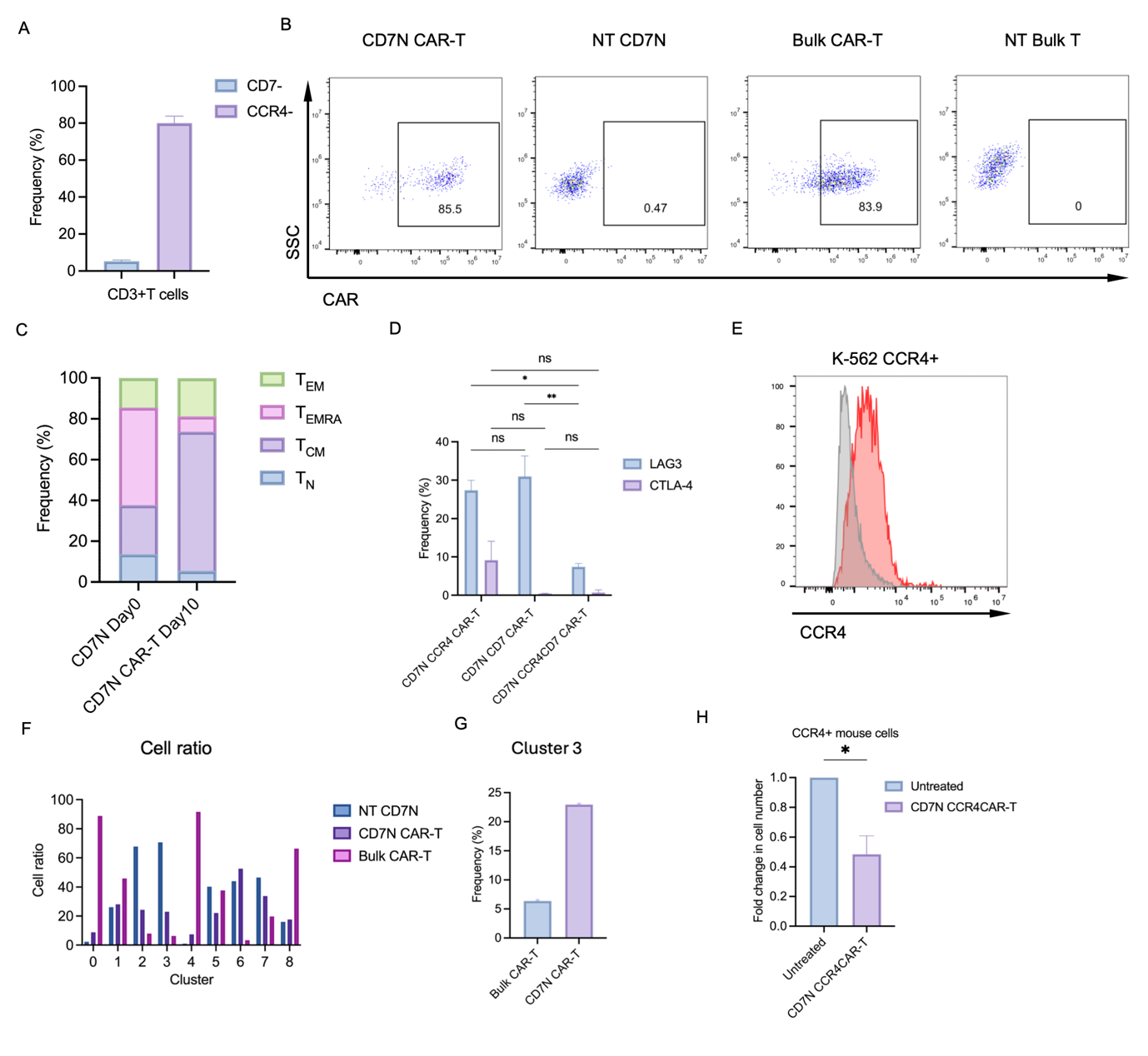


**Supplementary Figure 1**

(A) Analysis of the expression of CD7 and CCR4 on CD3+T cells. Data are presented as mean ± SEM from six donors.

(B) Representative dot plots illustrating CAR expression on Bulk CAR-T, CD7N CAR-T, NT CD7N and NT Bulk T cells during the culture process.

(C) Analysis of the memory phenotype of CD7N CAR-T cell populations on day 0 and day 10. Data are presented as the average from three donors.

(D) Expression of immune checkpoint receptors on CD7N CAR-T, CD7N CCR4CAR-T and CD7N CD7CAR-T, assessed by flow cytometry on day 10. The results are shown as mean ± SEM (n = 3). *p < 0.05, **p < 0.01, two-way ANOVA.

(E) CCR4 expression in CCR4+transiently transfected K-562 cells was assessed by flow cytometry and compared with wild-type K-562 cells.

(F) Proportional distribution of nine clusters across NT CD7N, CD7N CAR-T, and Bulk CAR-T cells.

(G) Comparison of cluster 3 proportions between CD7N CAR-T and Bulk CAR-T groups.

(H) Relative fold change in the number of CCR4+ mouse T cells after 24 hours of co-culture with CD7N CCR4 CAR-T cells compared to control cells (without CAR-T cells) at an E:T ratio of 0.8:1. Results are presented as mean ± SEM (n=3).


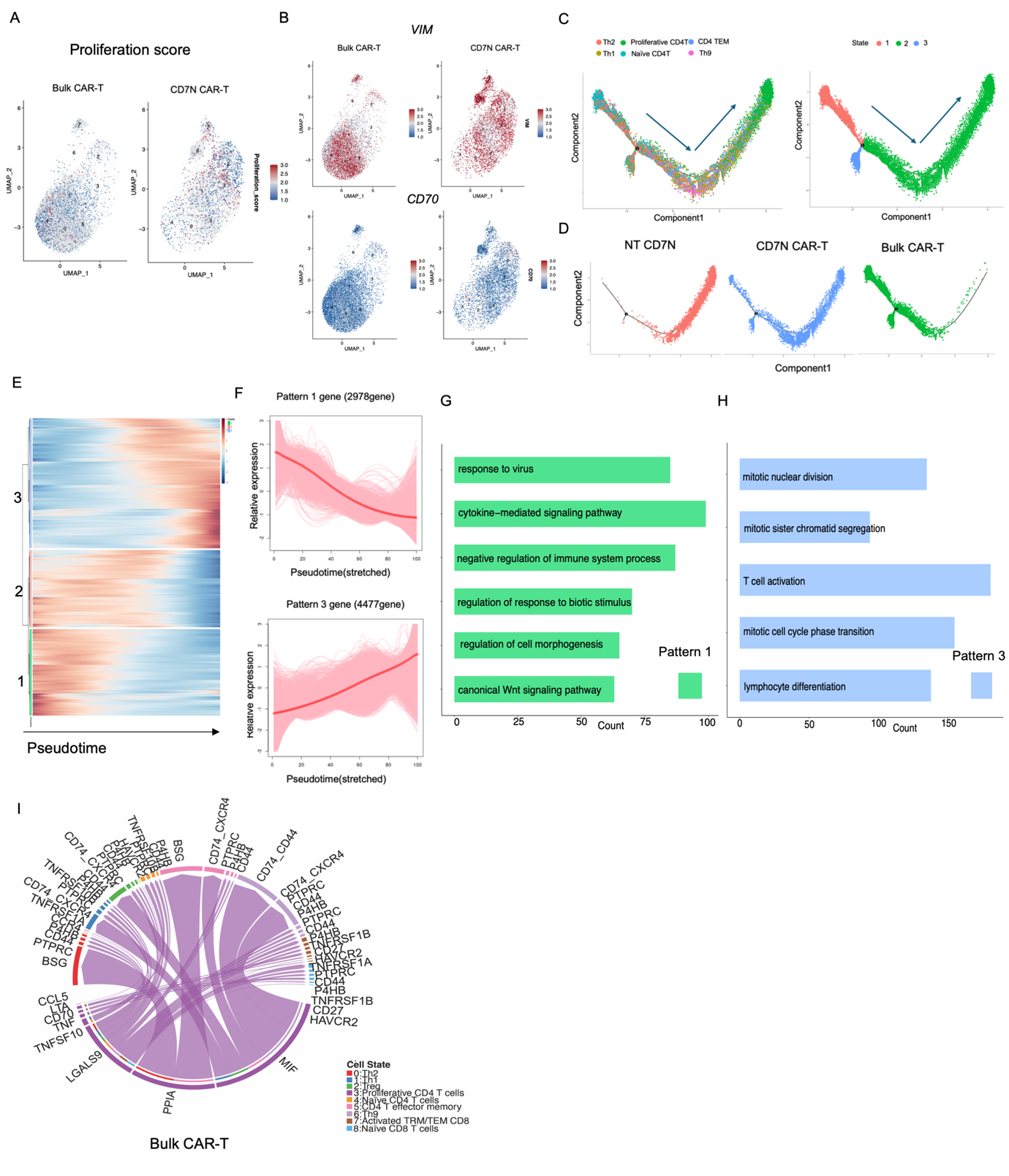


**Supplementary Figure 2**

(A) Distribution of proliferation scores and (B) expression patterns of *VIM* and *CD70* gene signatures visualized on the UMAP plot.

(C) Pseudotime estimation and three inferred trajectory states generated using Monocle2, with random cells from naïve CD4 T cells designated as roots.

(D) The Pseudotime values and inferred trajectories for CD7N CAR-T, Bulk CAR-T and NT CD7N.

(E) A heatmap was generated to display the DEGs across three spatially defined clusters during the pseudotime trajectory. Functional annotation of these clusters was performed using GO enrichment analysis, resulting in the identification of three distinct functional groups.

(F) Both panels display the expression profiles of genes belonging to patterns 1 and 3, which align with the inferred trajectory progressing toward the terminal differentiation state.

(G-H) Bar charts highlight the primary biological processes associated with functional pattern 3, as identified in the DEG heatmap, which showed activation during the pseudotime trajectory. Conversely, biological processes associated with pattern 1 also appear to be repressed.

(I) L–R interactions originating from cluster 3 were identified within the Bulk CAR-T group.
